# Supplementary material for: Understanding Hematopoietic Stem Cell Development through Functional Correlation of Their Proliferative Status with the Intra-aortic Cluster Architecture
Source: Stem Cell Reports. 2017 May 4;8(6):1549–62. doi: 10.1016/j.stemcr.2017.04.003 (PMC5469869; doi:10.1016/j.stemcr.2017.04.003)
Supplement: Document S1. Figures S1–S4 and Tables S1 and S2 [file mmc1.pdf]

Stem Cell Reports, Volume *8*

Supplemental Information

Understanding Hematopoietic Stem Cell Development through Functional Correlation of Their Proliferative Status with the Intra-aortic Cluster Architecture

Antoniana Batsivari, Stanislav Rybtsov, Celine Souilhol, Anahi Binagui-Casas, David Hills, Suling Zhao, Paul Travers, and Alexander Medvinsky

Supplementary Figure 1. Related to Figure 1.

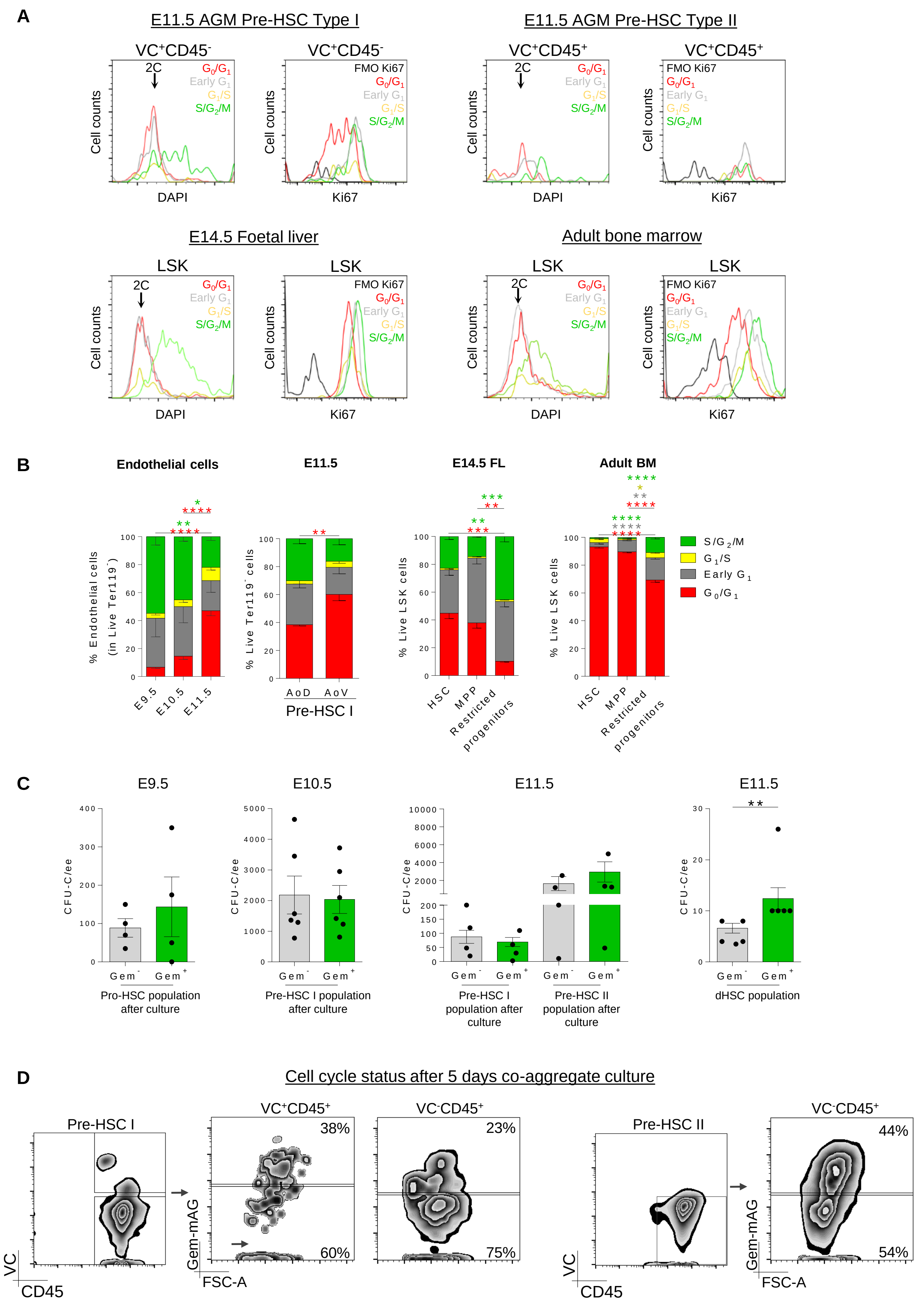

Supplementary Figure 1. Related to Fig. 1. **Validation of Fucci reporters and cell cycle analysis of haematopoietic populations.**

(A) Validation of the Fucci reporters (Geminin-mAG and Cdt1-mKO2) in haematopoietic populations at various stages with Ki67 and DAPI staining (2C, diploid DNA) (2-3 independent experiments). All populations are gated on Live Lineage^{-} cells. (B) Flow cytometry of various populations of Fucci embryos and adults (3-4 independent experiments). The erro r bars show SEM. The endothelial population is identified as $VC^{+}CD45^{-}CD41^{-}CD43^{-}$ and the pre-HSC Type I population as $VC^{+}CD45^{-}CD41^{-}CD43^{+}$, in the AGM region. The HSC population is identified as LSK $CD150^{+}CD48^{-}$, the MPP as LSK $CD150^{-}CD48^{-}$ and the restricted progenitors as LSK $CD150^{-}CD48^{+}$, both in FL and BM. (C) In vitro methylcellulose assay of pro/pre-HSC sorted populations after co-aggregate culture and dHSC populations directly after sorting (at least 4 independent experiments). The error bars show SEM . (D) Representative flow cytometry plots of E11.5 pre-HSC I and II sorted populations after 5 days co-aggregate culture (at least 3 independent experiments). Geminin-mAG$^{-/+}$ fractions had no difference in their output in terms of cell cycle status. AoV, ventral part of dorsal aorta,; AoD, Dorsal part of dorsal aorta; FL, foetal liver; BM, bone marrow. **p<0.00 5, ***p<0.0005, ****p<0.0001 .

Supplementary Figure 2. Related to Figures 2 and 3

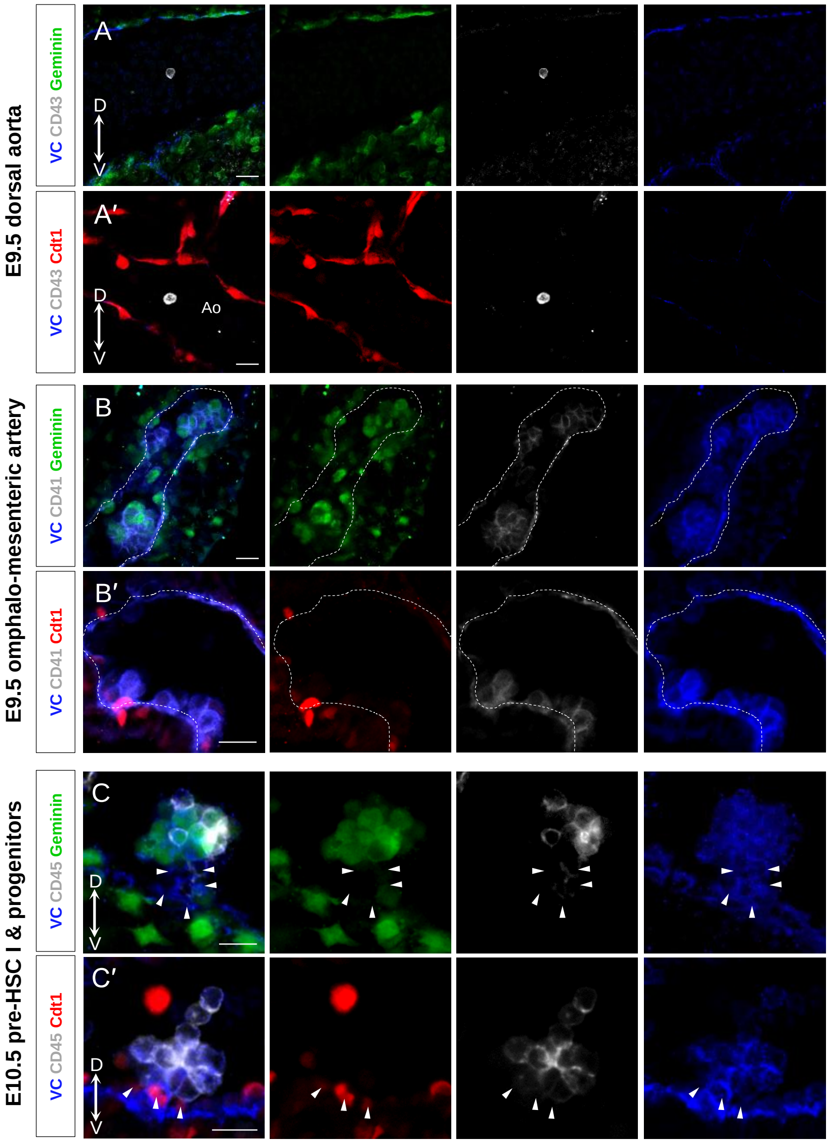

Supplementary Figure 2. Related to Fig. 2, 3. **Cell cycle status of E9.5 and E10.5 HSC precursors.**

(A), (B), (C) Geminin-mAG and (A′), (B′), (C′) Cdt1-mKO2 embryos stained for different haematopoietic markers. (A), (A′) CD43 is not expressed in the dorsal aorta of E9.5 embryos. (B), (B′) CD41 is expressed in the E9.5 OMA cell clusters, which are Geminin-mAG^{+}Cdt1-mKO2^{-}. (C), (C′) CD45 is localised at the top of the E10.5 intra-aortic clusters and marks mainly progenitors that are actively cycling (Geminin-mAG^{+}Cdt1-mKO2^{-}). White arrowheads show the G_0/G_1 (Geminin-mAG^{-}Cdt1-mKO2^{+}) cells that CD45^{-}. Scale bar, 15 µm.

Supplementary Figure 3. Related to Figure 3.

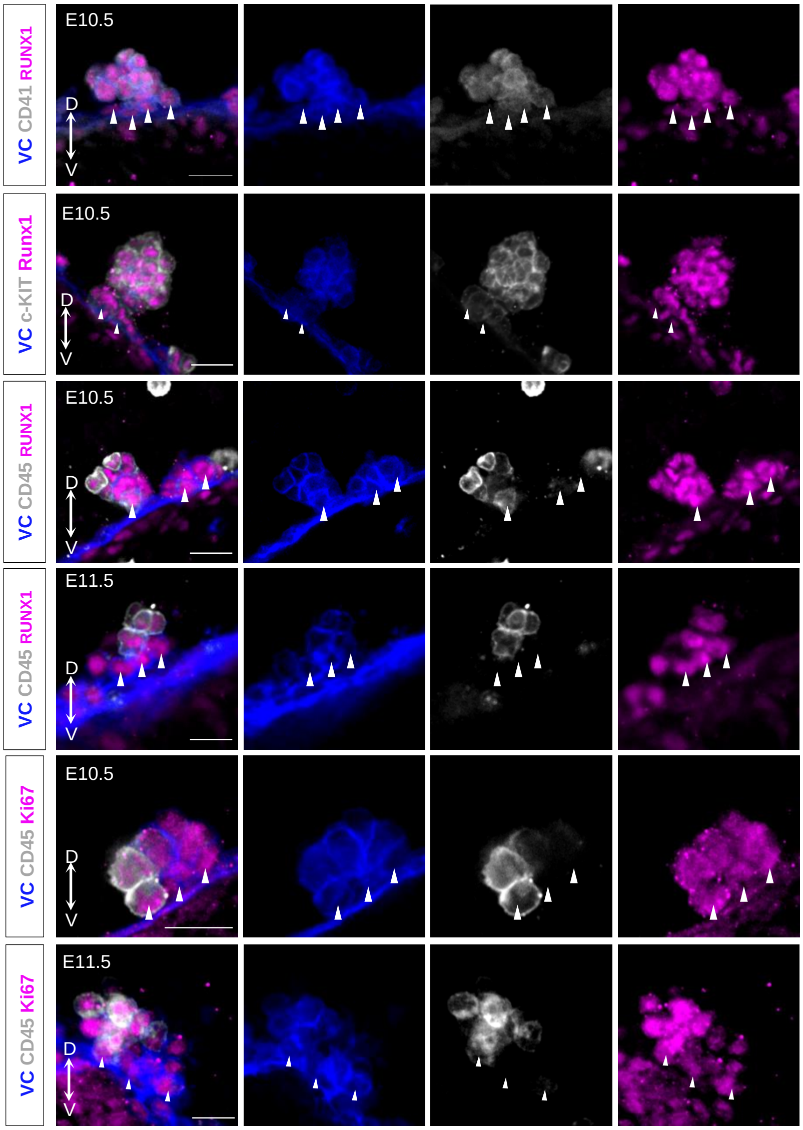

Supplementary Figure 3. Related to Fig. 3 & 4. **Characterisation of intra-aortic clusters with various haematopoietic markers.**

Representative images of wild type E10.5-11.5 embryos showing the expression pattern of RUNX1, CD41, c-KIT, CD45 and Ki67 within intra-aortic clusters. Of note, the cells at the base of these clusters are haematopoietic (white arrowheads). Scale bar, 15 µm.

Supplementary Figure 4. Related to Figure 3 and 4.

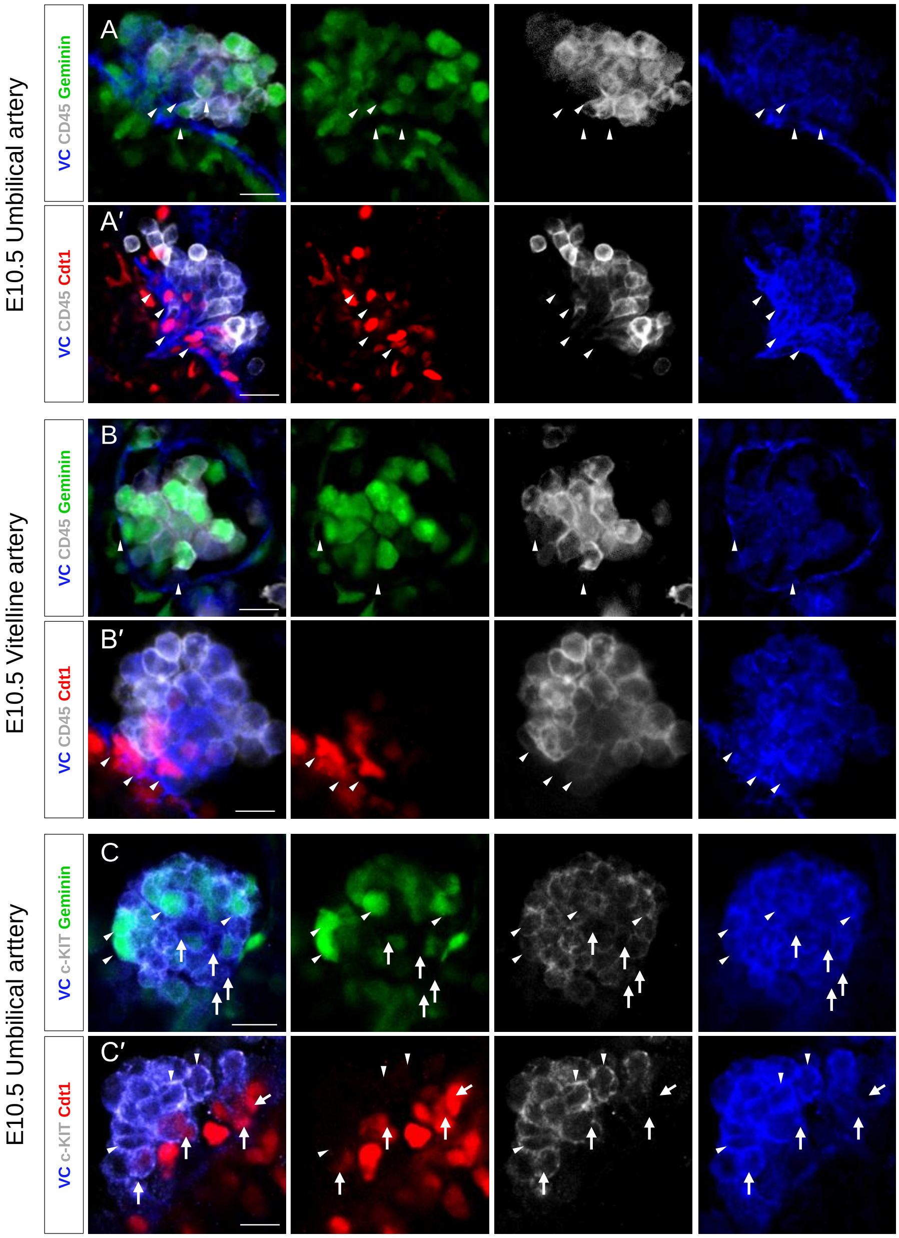

Supplementary Figure 4. Related to Fig. 3 & 4. **Cell cycle status of haematopoietic clusters in the extra-embryonic vessels.** (A), (B), (C) Geminin-mAG and (A′), (B′), (C′) Cdt1-mKO2 embryos stained for different haematopoietic markers. (A), (A′), (B), (B′) Representative images of haematopoietic clusters with slowly cycling cells (Geminin-mAG^{-}Cdt1-mKO2^{+}) at their base in umbilical and vitelline arteries (white arrowheads) in E10.5 embryos. (C), (C′) c-KIT expression in umbilical artery clusters showing that c-KIT^{lo} cells are Geminin-mAG^{-}Cdt1-mKO2^{+} (white arrows), while c-KIT^{hi} expressing cells are Geminin-mAG^{+}Cdt1-mKO2^{-} (white arrowheads). Scale bar, 10µm.

Supplementary Table 1. Related to Figure 1.

A

**Experiment**	**No. cells/ee (1 aggregate)**		**No. cells/aggregate after culture**		**Dose transplanted/ recipient**
	Gem-mAG$^-$	Gem-mAG$^+$	Gem-mAG$^-$	Gem-mAG$^+$	
E9.5 pro-HSC Expt.1	130	36	66612	41244	4 ee
E9.5 pro-HSC Expt.2	32	20	65743	43956	5 ee
E10.5 pre-HSC I Expt.1	117	62	1231984	1060184	4 ee
E10.5 pre-HSC I Expt.2	131	56	934617	1226718	4 ee
E10.5 pre-HSC I Expt.3	181	91	1513570	892790	4 ee
E11.5 pre-HSC I Expt.1	1209	1041	43850	65710	1 ee
E11.5 pre-HSC I Expt.2	1475	960	39840	33010	1 ee
E11.5 pre-HSC I Expt.3	1381	314	29050	23710	1 ee
E11.5 pre-HSC I Expt.4	1346	935	48213	37854	1 ee
E11.5 pre-HSC I Expt.5	1399	521	26084	30588	1 ee
E11.5 pre-HSC II Expt.1	397	1041	69312	131835	1 ee
E11.5 pre-HSC II Expt.2	437	578	70966	87188	1 ee
E11.5 pre-HSC II Expt.3	794	332	94278	125761	1 ee
E11.5 pre-HSC II Expt.4	1143	771	80580	93525	1 ee
E11.5 pre-HSC II Expt.5	716	411	79890	89050	1 ee

B

**Experiment**	**No. cells transplanted/recipient**			
	Gem-mAG$^-$		Gem-mAG$^+$	
E11.5 dHSC Expt.1	20487		10471	
E11.5 dHSC Expt.2	45567		29463	
E11.5 dHSC Expt.3	32467		12610	
E11.5 dHSC Expt.4	102000		84500	
E11.5 dHSC Expt.5	182909		53090	
E11.5 dHSC Expt.6	137181		39181	
	G_0/G_1	Early G_1	G_1/S	$S/G_2/M$
E14.5 FL Expt.1	342	465	222	414
E14.5 FL Expt.2	455	426	356	453
E14.5 FL Expt.3	34	36	5	46
Adult BM Expt.1	6417	1839	183	1204
Adult BM Expt.2	1287	116	61	46

Supplementary Table 1. Related to Figure 1. **Cell numbers for co-aggregate culture and transplantations.** (A) E9.5, 10.5 and 11.5 Geminin-mAG reporter embryos were sorted for pro/pre-HSCs. Representative numbers of sorted cells used for one co-aggregate (1 ee), representative total cell numbers of one co-aggregate after culture and the dose transplanted in each experiment are shown in the table. (B) The number of sorted cells from E11.5 AGM region (Geminin-mAG reporter embryos), foetal liver and adult bone marrow (Fucci embryos and adults) that were transplanted directly in recipients is shown in this table.

Supplementary Table 2. Related to Figure 4.

A

**Correlation Analysis c-KIT levels vs cell cycle status fractions of pre-HSC I population**					
	**cKIT vs G_0-G_1** (red)	**cKIT vs early G_1** (grey)	**cKIT vs G_1/S** (yellow)	**cKIT vs S/G_2/M** (green)	**cKIT vs ratio red/green**
**r**	-0.8	-0.5	-0.4	-0.1	-0.7
**R square**	0.7	0.3	0.1	0.02	0.5
**P value (two-tailed)**	0.0004	0.04	0.2	0.7	0.004
**P value summary**	***	*	ns	ns	**

B

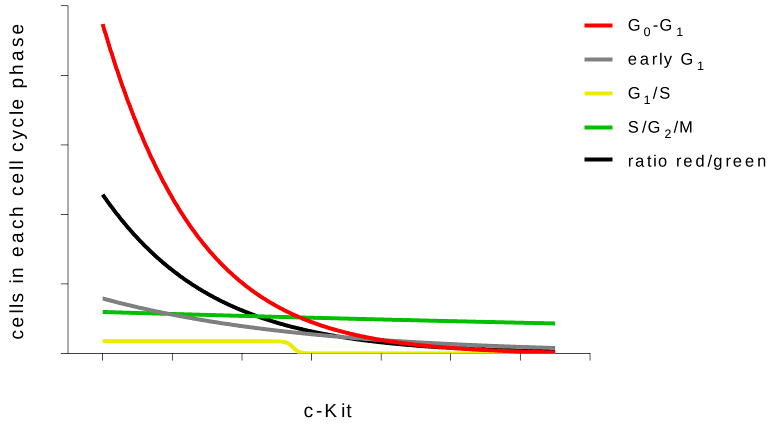

Supplementary Table 2. Related to Figure 4. **Correlation analysis of c-KIT levels with different cell cycle fractions within the pre-HSC Type I population.**

(A) E11.5 Pre-HSC Type I population from double transgenic Fucci embryos was analysed for cell cycle status and c-KIT levels by flow cytometry. Cell numbers extracted from the FlowJo analysis were used for the correlation analysis performed with GraphPad. The table shows the correlation coefficient (r), the r square and the p value (see Experimental Procedures). Pre-HSC Type I cells in G_0/G_1 inversely correlate with c-KIT levels similar to cells in early G_1 phase. Also the ratio of (G_0/G_1 cells) / (S/G_2/M cells) negatively correlate with c-Kit levels; therefore when c-KIT levels increase, the probability of G_0/G_1 cells within this fraction diminishes. On the contrary, there is no correlation between cells found in G_1/S and S/G_2/M phases and c-KIT levels and thus change in one variable (i.e. cell cycle phase) does not cause change in the other variable (i.e. c-Kit levels). (B) Nonlinear regression lines fit these data and depict the negative correlation between slowly cycling (G_0/G_1 and early G_1) cells and c-KIT levels.
