# Supplementary material for: Understanding Hematopoietic Stem Cell Development through Functional Correlation of Their Proliferative Status with the Intra-aortic Cluster Architecture
Source: Stem Cell Reports. 2017 May 4;8(6):1549–62. doi: 10.1016/j.stemcr.2017.04.003 (PMC5469869; doi:10.1016/j.stemcr.2017.04.003)
Supplement: Document S2. Article plus Supplemental Information [file mmc2.pdf]

Stem Cell Reports
**Article**

ISSCR

OPEN ACCESS

# Understanding Hematopoietic Stem Cell Development through Functional Correlation of Their Proliferative Status with the Intra-aortic Cluster Architecture

Antoniana Batsivari,[1] Stanislav Rybtsov,[1] Celine Souilhol,[2] Anahi Binagui-Casas,[1] David Hills,[1] Suling Zhao,[1] Paul Travers,[1] and Alexander Medvinsky[1,*]

[1]Institute for Stem Cell Research, Medical Research Council Centre for Regenerative Medicine, University of Edinburgh, SCRM Bioquarter, 5 Little France Drive, Edinburgh EH16 4UU, UK

[2]Department of Infection, Immunity & Cardiovascular Disease, University of Sheffield, Sheffield S10 2RX, UK

*Correspondence: a.medvinsky@ed.ac.uk

http://dx.doi.org/10.1016/j.stemcr.2017.04.003

## SUMMARY

During development, hematopoietic stem cells (HSCs) emerge in the aorta-gonad-mesonephros (AGM) region through a process of multi-step maturation and expansion. While proliferation of adult HSCs is implicated in the balance between self-renewal and differentiation, very little is known about the proliferation status of nascent HSCs in the AGM region. Using Fucci reporter mice that enable in vivo visualization of cell-cycle status, we detect increased proliferation during pre-HSC expansion followed by a slowing down of cycling once cells start to acquire a definitive HSC state, similar to fetal liver HSCs. We observe time-specific changes in intra-aortic hematopoietic clusters corresponding to HSC maturation stages. The proliferative architecture of the clusters is maintained in an orderly anatomical manner with slowly cycling cells at the base and more actively proliferating cells at the more apical part of the cluster, which correlates with c-KIT expression levels, thus providing an anatomical basis for the role of SCF in HSC maturation.

## INTRODUCTION

The AGM region plays an important role in development of HSCs that give rise to the adult hematopoietic system (Kumaravelu et al., 2002; Medvinsky and Dzierzak, 1996; Müller et al., 1994; Medvinsky et al., 2011; Ciau-Uitz et al., 2016). The pool of immature precursors (pre-HSCs), which cannot yet repopulate adult irradiated recipients, gradually expands and matures in the AGM region (Rybtsov et al., 2016). This concealed dramatic expansion of pre-HSCs culminates in the emergence of a few definitive (d)HSCs in the E11 AGM region followed by a sudden increase in their number in the E12 fetal liver, detectable by direct transplantation into adult irradiated recipients (Kumaravelu et al., 2002; Ema and Nakauchi, 2000; Rybtsov et al., 2016).

Cell proliferation is one of critical factors involved in many developmental processes (Budirahardja and Gönczy, 2009; Lange and Calegari, 2010; Kaldis and Richardson, 2012), and the proliferative status of adult HSCs is an important feature of their biology. In the fetal liver, HSCs expand, probably through symmetric division until week 3–4 postnatally, then become quiescent (Bowie et al., 2006). Proliferative quiescence in the adult maintains "stemness" of HSCs and prevents their exhaustion (Passegué et al., 2005; Wilson et al., 2008; Seita and Weissman, 2010; Takizawa et al., 2011; Pietras et al., 2011; Nakamura-Ishizu et al., 2014). Physiological demands drive HSCs to enter proliferation, while a balance is maintained to ensure HSC self-renewal and differentiation. The bone marrow niche maintains HSC quiescence through essential signaling (Jude et al., 2008; Mendelson and Frenette, 2014; Morrison and Scadden, 2014). By contrast, downstream committed progenitors, which are involved in the immediate production of mature blood cells, are significantly more proliferative (Passegué et al., 2005).

Given the importance of proliferation in cell commitment and differentiation, here we have studied proliferative changes during HSC maturation steps, which to date have not been studied in detail. We showed previously that in culture developing HSCs of the AGM region proliferate slower than committed progenitors (Taoudi et al., 2008). More recent in vivo analysis of the dramatic pre-HSC expansion in the AGM region suggests that proliferation or/and cell recruitment may play a role (Rybtsov et al., 2016).

In vitro modeling has proved to be a powerful and informative approach for the identification of pre-HSC states and dissection of HSC developmental mechanisms (Taoudi et al., 2008). HSCs develop through a multi-step process: pro-HSC → pre-HSC I → pre-HSC II → dHSC, which involves sequential upregulation of hematopoietic markers CD41 (Itga2b), RUNX1 (AML1), CD43 (Spn), and CD45 (Ptprc) in VE-CADHERIN$^+$ (VC) precursors (Rybtsov et al., 2011, 2014; Taoudi et al., 2008; Medvinsky and Dzierzak, 1996; Liakhovitskaia et al., 2014; Swiers et al., 2013; Yoder et al., 1997). Pro-HSCs (VC$^+$CD41$^{lo}$CD43$^-$CD45$^-$) emerge at embryonic day 9.5 (E9.5), pre-HSCs type I (VC$^+$CD41$^{lo}$CD43$^+$CD45$^-$) at E10.5, and pre-HSCs type II (VC$^+$CD41$^{lo}$CD43$^+$CD45$^+$) at E11.5 stages. Low dHSC

CrossMark


© 2017 The Authors.
This is an open access article under the CC BY license (http://creativecommons.org/licenses/by/4.0/).

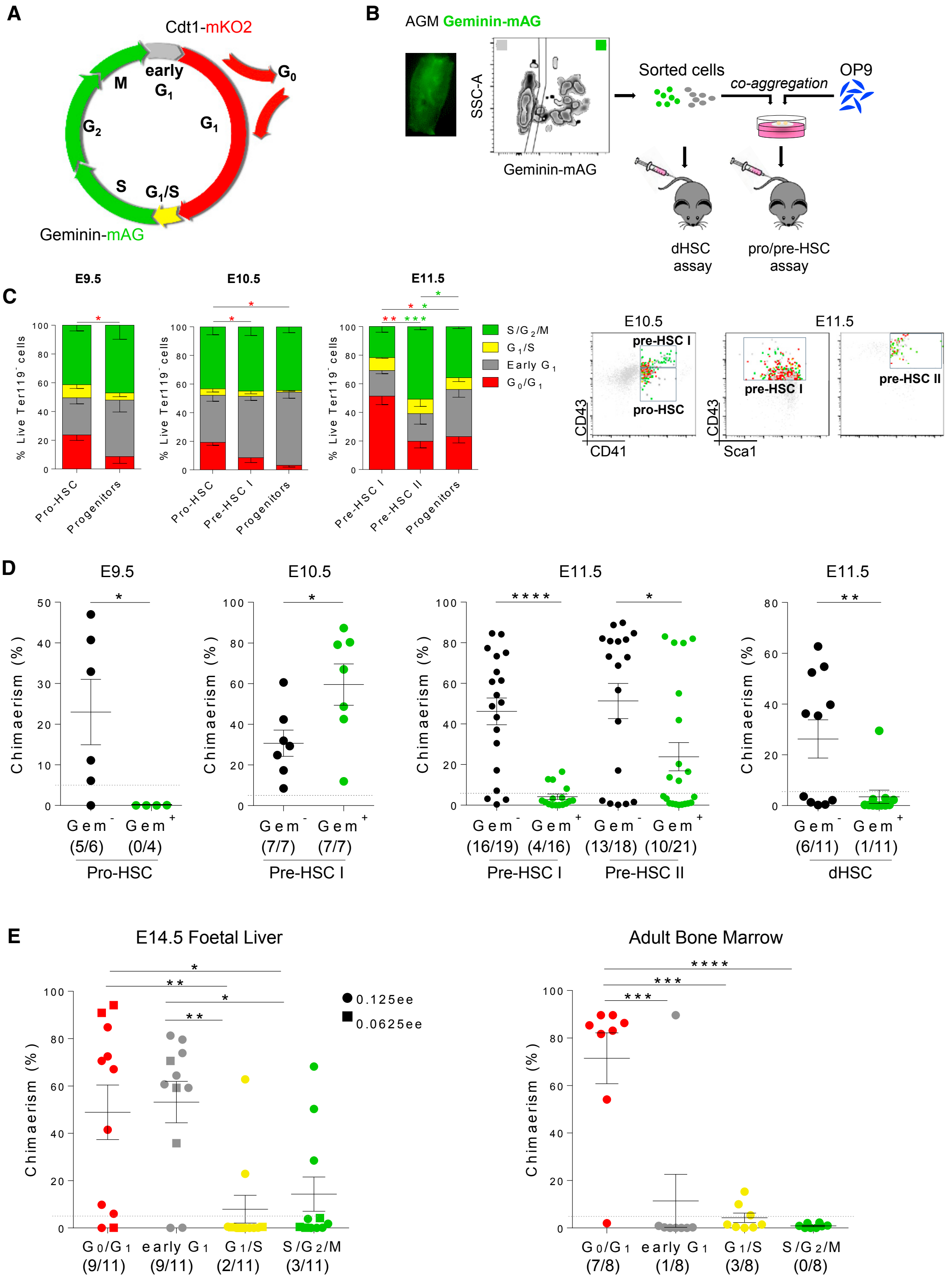


**Figure 1. Changes in Proliferative Status of Developing HSCs**

(A) Representation of cell-cycle analysis by Fucci reporters.

(B) Experimental design of transplantation assays of pro-/pre-HSCs and dHSCs.

(C) Flow-cytometry analysis of HSC precursors and committed progenitors in Fucci embryos at different developmental stages (three independent experiments) shown by bar graphs (error bars show SEM) and representative dot plots. All the populations analyzed are gated

(legend continued on next page)

numbers emerge at E11.5 and, although phenotypically similar to pre-HSCs type II, they can be detected by direct transplantations into irradiated recipients. Pro-/pre-HSCs have been identified in hematopoietic clusters budding from the endothelium of major embryonic arteries (Rybtsov et al., 2011, 2014; Taoudi et al., 2008; Yokomizo and Dzierzak, 2010; Kissa and Herbomel, 2010; Boisset et al., 2011; Gordon-Keylock et al., 2013; Ciau-Uitz et al., 2016).

Functional assessment of cell proliferation in live cells often involves Hoechst staining, which can be toxic and can alter the experimental outcome (Parish, 1999). Instead, we used the fluorescent ubiquitination-based reporter (Fucci) system, which enables noninvasive in vivo visualization of the cell-cycle status and their isolation for functional analysis (Sakaue-Sawano et al., 2008; Yo et al., 2015; Zielke and Edgar, 2015).

We describe here that pro-HSCs (at E9.5) initially slowly cycle, then enter active proliferation during E10.5–E11.5, which correlates with the expansion of the pro-/pre-HSC pool (Rybtsov et al., 2016). However, this phase is followed by gradual slowing down of proliferation, the first signs of which can be already observed in AGM dHSCs, in keeping with gradual acquisition of adult status by dHSCs. We also describe the orderly architectural evolvement of intra-aortic clusters in which stepwise HSC maturation and proliferation are linked. It is suggested that the proliferative pattern within the cluster is defined by c-KIT/SCF signaling.

## RESULTS

### Changes in Proliferative Status of Developing HSCs

To analyze the proliferative status of developing HSCs, we used the Fucci dual reporter mouse lines (see Experimental Procedures) appropriate for analysis of the hematopoietic system (Yo et al., 2015; Zielke and Edgar, 2015). Two anti-phase oscillating proteins that mark cell-cycle transitions Cdt1 (genetically labeled by mKO2; red fluorescence) and Geminin (genetically labeled by mAG; green fluorescence), which are controlled by the cell-cycle machinery through proteasomal degradation, have been used here as reporters. Cdt1-mKO2 is expressed during $G_0$ and $G_1$ phases and the cells fluoresce red, while Geminin-mAG (Gem-mAG) marks green the cells that are in $S/G_2/M$ phases (Figures 1A and S1A). During the $G_1/S$ transition cells become yellow and no reporter is expressed in early $G_1$ phase (shown as gray) (Figures 1A and S1A). Therefore, slowly cycling populations are represented mainly by red (Cdt1-mKO2$^+$) cells and actively cycling populations are mainly green (Gem-mAG$^+$) cells.

Flow-cytometry analysis of Fucci reporter embryos showed that in the caudal part of the E9.5 embryo endothelial cells were mainly within $S/G_2/M$ and early $G_1$ phases, indicating that they were actively proliferative. Only 6.6% (±1.6%) of the endothelial population (VC$^+$CD45$^-$CD41$^-$CD43$^-$) were found in the $G_0/G_1$ phases of the cell cycle (versus 54.8% ± 11.9% in $S/G_2/M$, $p = 0.0002$) (Figure S1B). By contrast, a significant proportion of cells in the pro-HSC population (VC$^+$CD45$^-$CD41$^{lo}$CD43$^-$) were in $G_0/G_1$ phases (23.7% ± 7.6% versus $G_0/G_1$ endothelial population, $p = 0.004$), suggesting that a fraction of these cells emerging from the endothelium slow down their cycling (Figure 1C). Compared with the pro-HSC population, more committed hematopoietic progenitors (VC$^-$CD45$^-$CD41$^{lo}$CD43$^+$) (Rybtsov et al., 2014) were actively proliferating (progenitors, 8.6% ± 9.3% versus pro-HSC, 23.7% ± 7.6% in $G_0/G_1$; $p = 0.02$) (Figure 1C). To functionally define the cell-cycle status of pro-HSCs, we sorted Gem-mAG$^+$ and Gem-mAG$^-$ fractions of this population, co-aggregated them with OP9 cells for 7 days in culture, and transplanted them into irradiated recipients, as described previously (Rybtsov et al., 2014) (Figure 1B). Only the Gem-mAG$^-$ fraction generated transplantable dHSCs, suggesting that pro-HSCs are slowly cycling (Figure 1D and Table S1). Meanwhile, both Gem-mAG$^+$ and Gem-mAG$^-$ fractions of the pro-HSC population were able to generate colonies of myeloid cells in methylcellulose CFU-C assays (Figure S1C).

While the E9.5 endothelium is mainly proliferating, this population slows down its cycle during the following days of development since the proportion of $G_0/G_1$ endothelial cells increases to 47% (±6.1) by E11.5 (versus E9.5: 6.6% ± 1.6%, $p < 0.0001$) (Figure S1B). In contrast to pro-HSCs observed in E9.5–10.5 embryos, the proportion of E10.5 pre-HSC type I (VC$^+$CD45$^-$CD41$^{lo}$CD43$^+$) in the $G_0/G_1$

on Live Ter119$^-$ cells. The pro-HSC population is identified as VC$^+$CD45$^-$CD41$^{lo}$CD43$^-$, while the progenitors are VC$^+$CD45$^-$CD41$^{lo}$CD43$^+$. The pre-HSC type I population is identified as VC$^+$CD45$^-$CD41$^{lo}$CD43$^+$, while the E10.5 progenitors are VC$^+$CD45$^+$. The pre-HSC type II population is identified as VC$^+$CD45$^+$CD41$^{lo}$CD43$^+$ and the E11.5 progenitors as VC$^-$CD45$^+$. *p < 0.05, **p < 0.01, ***p < 0.005.
(D) Transplantation assays of pro-/pre-HSCs and dHSCs after sorting on the basis of the Geminin-mAG reporter (at least three independent experiments). *p < 0.05, **p < 0.01, ****p < 0.0001.
(E) Transplantation assays of fetal liver (LSK CD48$^-$) HSCs and bone marrow (LSK CD150$^+$CD48$^-$) HSCs after sorting on the basis of the Fucci reporters (2–3 independent experiments). The dashed line shows the 5% chimerism threshold and the error bars show SEM. *p = 0.02, **p = 0.005, ***p = 0.0006, ****p < 0.0001.
See also Figure S1 and Table S1.

phases decreased to 8.5% (±6.7%) (versus E10.5 pro-HSC: 19.2% ± 4%, p = 0.03), suggesting that by this stage the HSC lineage becomes more proliferative (Figure 1C). Indeed, functional validation using ex vivo maturation and transplantation showed that in contrast to E9.5 pro-HSCs, pre-HSC type I resided in both the Gem-mAG$^+$ and Gem-mAG$^-$ fraction, which is in line with the dramatic expansion of the pre-HSC pool at E10.5 (Figure 1D and Table S1) (Rybtsov et al., 2016). We observed higher repopulation levels from the Gem-mAG$^+$ fraction but no bias in multi-lineage differentiation compared with the Gem-mAG$^-$ fraction (data not shown). Similar to the E9.5 pro-HSC population, both Gem-mAG$^+$ and Gem-mAG$^-$ fractions of E10.5 pre-HSC type I were equally capable of generating myeloid colonies in the methylcellulose (Figure S1C).

By E11.5, immunophenotypic analysis showed a dramatic increase of $G_0/G_1$ cells in the pre-HSC type I population compared with E10.5, from 8.5% ± 6.7% to 51.3% ± 10.3% (p = 0.02), respectively (Figure 1C). By contrast, the more advanced pre-HSC type II population (VC$^+$CD45$^+$CD41$^{lo}$ CD43$^+$Sca1$^+$) was found mainly in early $G_1$ and S/$G_2$/M phases associated with active cell cycling (19.3% ± 12.8% and 50.7% ± 3.7%, respectively versus $G_0/G_1$: 19.7% ± 7.9%, p = 0.02) (Figure 1C). Previous analysis showed that pre-HSCs emerge predominantly in the ventral domain of the dorsal aorta (AoV) with some contribution from the dorsal domain (AoD) (Souilhol et al., 2016; Taoudi and Medvinsky, 2007). Interestingly, immunophenotypic analysis at E11.5 revealed a larger proportion of the pre-HSC type I population in S/$G_2$/M phases from the AoD compared with AoV (30% ± 7.1% versus 16.1% ± 8.7%, respectively; p = 0.02), which is reminiscent of committed progenitor cells (Figure S1B). By contrast, no proliferative difference was observed between AoV- and AoD-derived endothelial or pre-HSC type II populations (data not shown).

A striking change in cell-cycle status was observed in pre-HSC type I by E11.5. Functional transplantations demonstrated that in contrast to E10.5, these cells resided almost exclusively in $G_0/G_1$, with only a few low repopulating cells residing in S/$G_2$/M phases (Figures 1D and S1D; Table S1). By contrast, pre-HSCs type II were found in both Gem-mAG$^+$ and Gem-mAG$^-$ fractions (Figure 1D). Notably, direct transplantations (without prior culturing) showed that mature E11.5 dHSCs were in $G_0/G_1$ phases, indicating that acquisition of the adult status is accompanied by reducing cycling (Figure 1D and Table S1). Although both E11.5 pre-HSCs and dHSCs were predominantly Gem-mAG$^-$, CFU-C were equally well represented by both Gem-mAG$^+$ and Gem-mAG$^-$ fractions (Figure S1C).

When we analyzed E14.5 fetal liver, we found that the majority of HSCs were also within $G_0/G_1$ or early $G_1$ phases ($G_0/G_1$: 44.8% ± 6.7%) and became quiescent in the adult bone marrow ($G_0/G_1$: 93.1% ± 1.4%) (Figure S1B), in contrast to restricted progenitors ($G_0/G_1$ 10.2% ± 0.8% in FL, p = 0.0005; $G_0/G_1$ 69.2.8% ± 2.6% in BM, p = 0.0002) (Figure S1B). These findings in combination with transplantation assays (Figure 1E and Table S1) are in line with previous reports (Bowie et al., 2006, 2007a; Passegué et al., 2005). Based on limiting dilution transplantation analysis (Figure 1E) (ELDA; Hu and Smyth, 2009), the numbers of fetal liver HSCs in $G_0/G_1$ phase were 17/100 cells and in S/$G_2$/M phases were 0.3/100 cells.

## Proliferative Structure of Intra-aortic Hematopoietic Clusters

HSCs and more committed progenitors develop in hematopoietic clusters that are budding from the endothelium of major arteries during E10.5 (Garcia-Porrero et al., 1995; North et al., 1999; Yokomizo and Dzierzak, 2010; Rybtsov et al., 2011; Boisset et al., 2015). Fucci mice allowed us to visualize the cell-cycle status within intra-aortic clusters. In the E9.5 embryo (caudal part), hematopoiesis occurs in two locations: pro-HSCs (CD41$^+$CD43$^-$) develop in the dorsal aorta and committed progenitors (CD41$^+$CD43$^+$) form a string of large cell clusters in the omphalomesenteric artery (OMA) (Zovein et al., 2010; Rybtsov et al., 2014). Hematopoietic cells are marked in these locations by the transcription factor Runx1, including flat cells integrated in the endothelial lining of the dorsal aorta (Figures 2A and 2A′) (Swiers et al., 2013; Rybtsov et al., 2014). We sought to identify candidate pro-HSCs using the Fucci reporters. We found a few VC$^+$RUNX1$^+$ cells, closely associated with the endothelium of the dorsal aorta, which are labeled by CD41 but not CD43 (Figures 2A, 2A′, S2A, and S2A′) and which were also Cdt1-mKO2$^+$ (Geminin-mAG$^-$) (Figures 2B and 2B′), characteristic of pro-HSCs identified by functional analysis (Figure 1D). It is conceivable that true low-expressing CD41+ pro-HSCs were not detectable using this immunofluorescence analysis. Our previous study showed that committed progenitors localized in the OMA are labeled by CD43 (Rybtsov et al., 2014). Indicative of their active proliferation, the majority of OMA CD43$^+$ cells were Geminin-mAG$^+$ (Cdt1-mKO2$^-$) both by flow cytometry (Figure 1C) and confocal analysis (Figures 2C, 2C′, S2B, and S2B′).

It is reasonable to assume that intra-aortic cell clusters are formed through budding from the endothelium of the dorsal aorta and may gradually build up through proliferation. Confocal analysis showed that the base of E10.5 clusters was VC$^+$CD41$^+$RUNX1$^+$ (and also c-KIT$^+$) but not CD43$^+$, and therefore harbored the most immature pro-HSC population (Figures 3B, 3B′, and S3). Notably, these cells closely associated with endothelium were in $G_0/G_1$ phases (Geminin-mAG$^-$/Cdt1-mKO2$^+$) (Figures 3A, 3B, and 3B′) as E9.5 pro-HSCs (Figure 1D). These $G_0/G_1$ cells at the base were

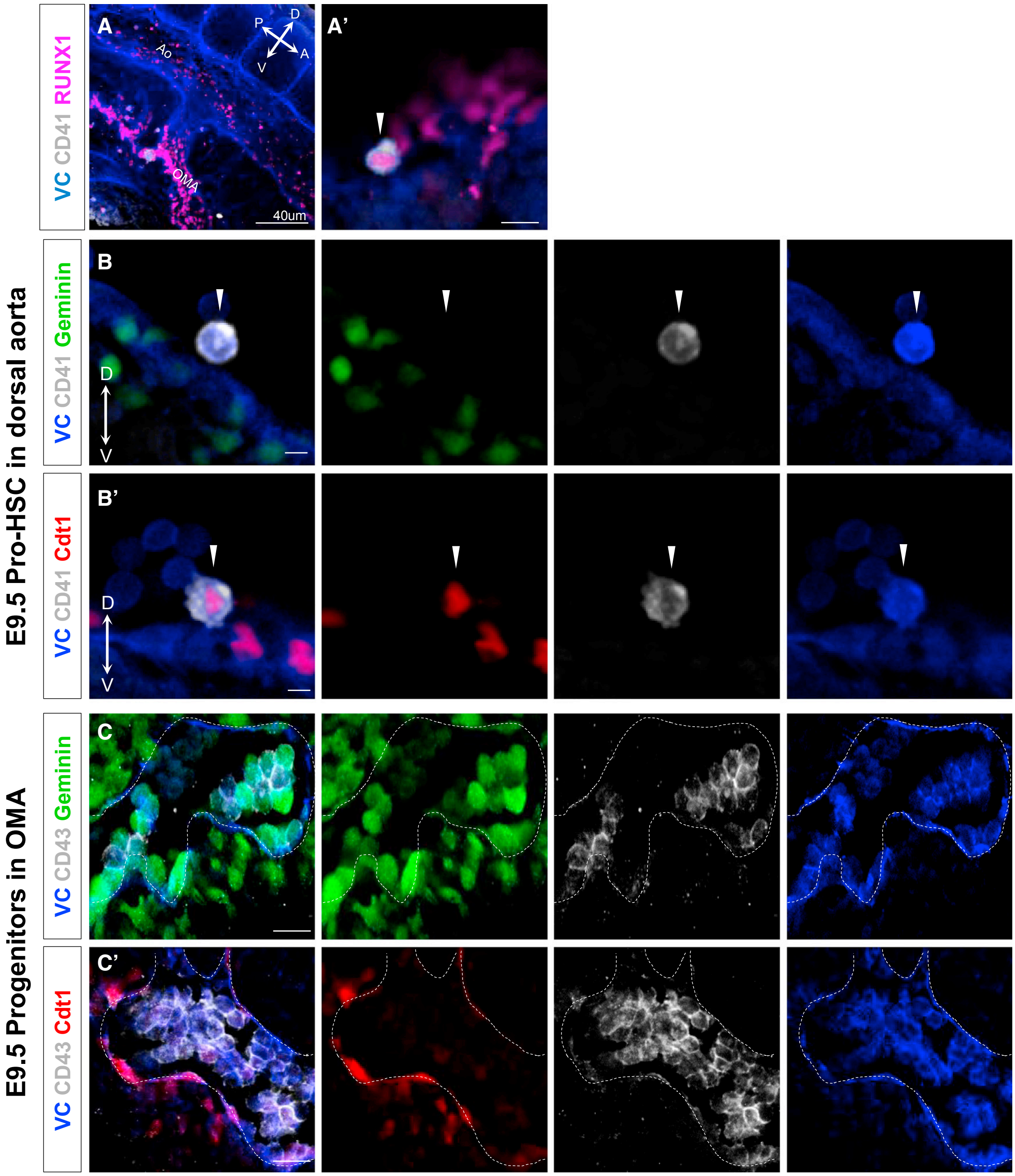


**Figure 2. Localization and Cell-Cycle Status of CD41$^{+}$Runx1$^{+}$ Cells in the E9.5 Embryo**

(A and A′) Wild-type E9.5 embryos stained against Runx1 and CD41. Low-magnification image (A) shows the Ao and OMA, while high magnification (A′) shows the localization of VC$^{+}$CD41$^{+}$Runx1$^{+}$ single cell in the dorsal aorta (white arrowhead) (at least three embryos).

(B) Geminin-mAG and (B′) Cdt1-mKO2 embryos stained against CD41 showing that pro-HSC phenotype cells (white arrowheads) in the dorsal aorta are in $G_0/G_1$ (at least three embryos).

(C) Geminin-mAG and (C′) Cdt1-mKO2 embryos stained against CD43 showing that progenitors (VC$^{+}$CD43$^{+}$) are specifically localized in OMA and they are Geminin-mAG$^{+}$Cdt1-mKO2$^{-}$ (at least three embryos). Dashed line shows the endothelium of the OMA.

Ao, dorsal aorta; OMA, omphalomesenteric artery; A, D, P, V, anterior, distal, posterior, ventral, respectively. Scale bars: (A) 40 μm; (A′, B, B′, C, C′) 10 μm. See also Figure S2.

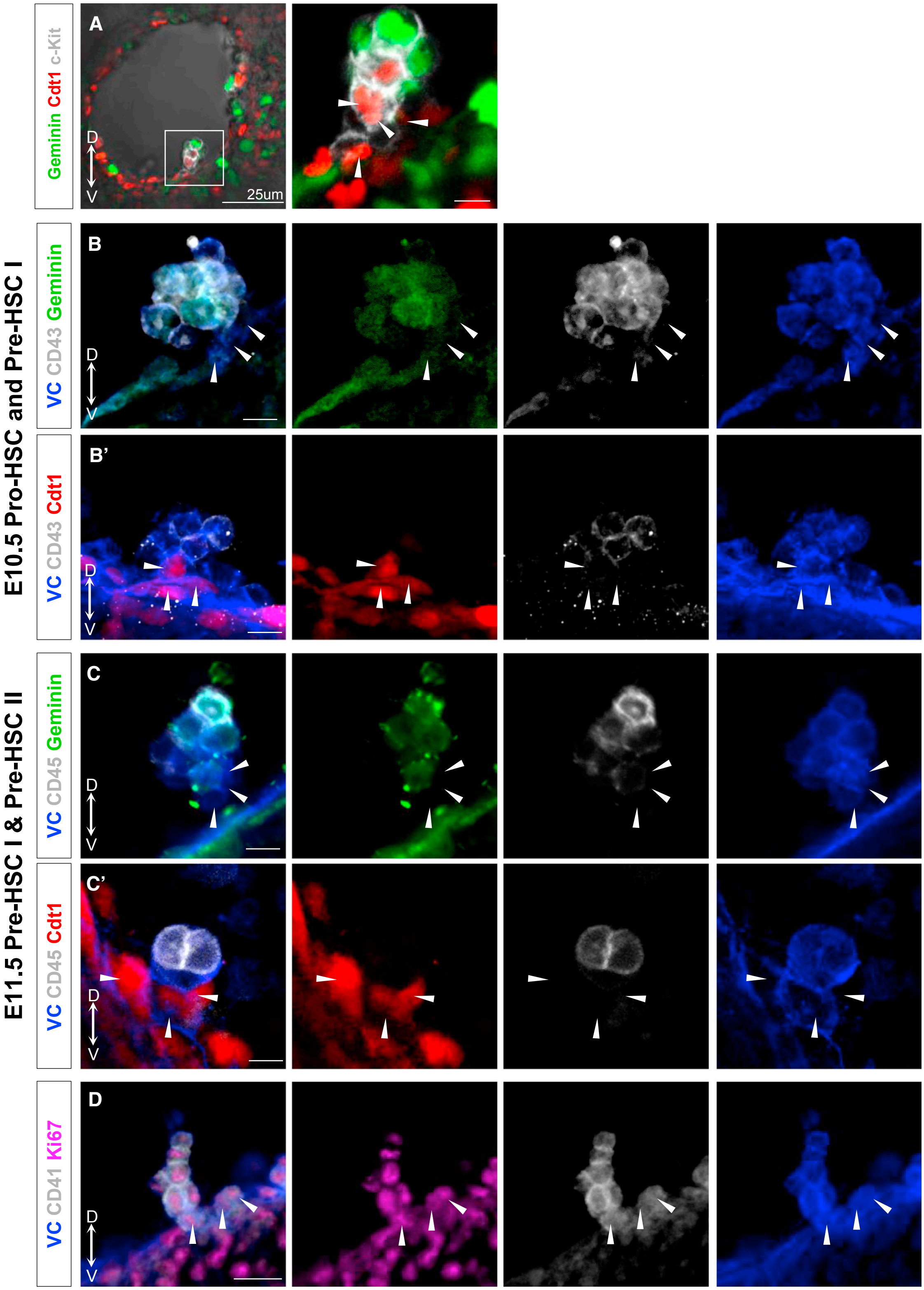


**Figure 3. Proliferative Structure of Intra-aortic Hematopoietic Clusters**

(A) E10.5 live section of Fucci embryo showing that cells at the base of the cluster are Geminin-mAG$^{-}$Cdt1-mKO2$^{+}$ (white arrowheads) (three embryos).

*(legend continued on next page)*

**Table 1. Proliferative Structure of Intra-aortic Hematopoietic Clusters**

| Sample | | No. of Clusters with VC$^+$CD45$^-$Cdt1$^+$ Base | Total No. of Clusters | % Clusters with Cdt1$^+$ Base |
|---|---|---|---|---|
| E10.5 | Embryo 1 | 12 | 12 | 100 |
| | Embryo 2 | 14 | 19 | 74 |
| | Embryo 3 | 21 | 24 | 87 |
| | Embryo 4 | 6 | 9 | 67 |
| E11.5 | Embryo 1 | 4 | 5 | 80 |
| | Embryo 2 | 12 | 15 | 80 |
| | Embryo 3 | 6 | 15 | 40 |
| | Embryo 4 | 12 | 14 | 89 |
| | Embryo 5 | 3 | 5 | 60 |

Intra-aortic clusters counted in E10.5 and E11.5 Cdt1-mKO2 embryos stained against CD45 and VC. The total number of clusters as well as the number/percentage of clusters with slowly cycling VC$^+$CD45$^-$Cdt1$^+$ base counted is shown.

observed in 67%–100% of clusters in four analyzed embryos (Table 1). Meanwhile, more apically located cells, presumably derived from the basal cells, were in S/G$_2$/M phases (Geminin-mAG$^+$/Cdt1-mKO2$^-$). Among these actively proliferating more apical cells were the VC$^+$CD43$^+$CD45$^-$ pre-HSC type I population and the committed VC$^+$CD43$^+$ CD45$^+$ progenitor population (note that pre-HSC type II are rare at this stage; Figures 3B, 3B′, S2C, and S2C′) (Rybtsov et al., 2011, 2014). Similar polarized basal-apical organization was observed in the usually significantly larger hematopoietic clusters of extra-embryonic (vitelline and umbilical) arteries (Figures S4A–S4B′).

By the next day (E11.5), when HSC precursors have matured further, we analyzed phenotypic changes in intra-aortic clusters. The base of E11.5 intra-aortic clusters was again represented by cells mainly in G$_0$/G$_1$ phases (Geminin-mAG$^-$/Cdt1-mKO2$^+$), which by this time upregulated CD43 and acquired a pre-HSC type I phenotype (CD45$^-$) (Figures 3C and 3C′), as observed in 40%–80% of clusters in individual embryos (n = 5) (Table 1). More apically located cells upregulated CD45$^+$ and thus acquired pre-HSC type II phenotype and, as expected from functional transplantation studies, were both Geminin-mAG$^+$ and Geminin-mAG$^-$ (Figures 3C and 3C′).

Although Fucci analysis allowed us to visualize key phases of the cell cycle in the developing HSC lineage, this does not explain whether the cells are resting or cycling. To address this issue, we used antibody staining for Ki67 and found that all cells in the cluster, including those which are at its base (Cdt1$^+$), were cycling (Figure 3D). Thus, Fucci analysis here reveals differences not between quiescent and cycling, but between slowly and rapidly cycling cells within the developing HSC lineage in the AGM region.

### c-KIT Expression Correlates with Cell-Cycle Status in HSC Precursors

The c-KIT/SCF signaling pathway is critically important for HSC development in the AGM region and in adult HSC niches (Rybtsov et al., 2014; Ding et al., 2012). Asymmetric, ventrally polarized expression of SCF in the AGM region correlates with predominant formation of intra-aortic clusters in the floor of the dorsal aorta (Souilhol et al., 2016). Our analysis of E10.5 clusters showed that c-KIT$^{low}$ cells in both intra-aortic and umbilical arteries were in G$_0$/G$_1$ phases, suggesting that they are slowly cycling (Figures 4A, 4A′, S4C, and S4C′, white arrows). Although we observed c-KIT$^{low}$ slowly proliferating cells in various positions, these were mostly localized to the base of the cluster, closely associated with endothelium (Figures 4A and 4A′). By contrast, high c-KIT levels correlated with actively proliferating Geminin-mAG$^+$ cells localized mainly apically in the cluster (Figures 4A, 4A′, S4C, and S4C′, white arrowheads). Accordingly, slowly cycling pre-HSCs type I (as shown functionally, Figure 1D, E11.5) were enriched for c-KIT$^{low}$ cells (G$_0$/G$_1$: 53.2% ± 7.4% are c-KIT$^{lo}$ versus 5.3% ± 1.7% are c-KIT$^{hi}$, p = 0.0006) compared with more actively cycling pre-HSC type II (G$_0$/G$_1$: 48.1% ± 3.3% are c-KIT$^{lo}$ versus 51.3% ± 3.2% are c-KIT$^{hi}$, p = not significant; S/G$_2$/M: 19.9% ± 2.6% are c-KIT$^{lo}$ versus 65.9% ± 4.9% are c-KIT$^{hi}$, $p < 0.0001$) (Figures 4B and Table S2), indicating that one of the roles of c-Kit/SCF signaling might be in expansion of the developing HSC pool (Rybtsov et al., 2016) through regulation of their proliferation (Bowie et al., 2007b; Sasaki et al., 2010; Shin et al., 2014).

## DISCUSSION

Cell proliferation plays an important role in various developmental processes. It underlies growth of tissues and organs and is involved in cell-fate decisions (Fuchs, 2009;

(B and C) Geminin-mAG and (B′, C′) Cdt1-mKO2 embryos stained for CD43 (B, B′) at E10.5 and CD45 (C and C′) at E11.5 to identify pro-/pre-HSCs (at least three embryos).
(D) Wild-type embryos stained against Ki67 (three embryos). White arrowheads show the cells at the base of the cluster.
Scale bars: (A) 25 μm; (B, B′, C, C′, D) 10 μm. See also Table 1 and Figures S2–S4.

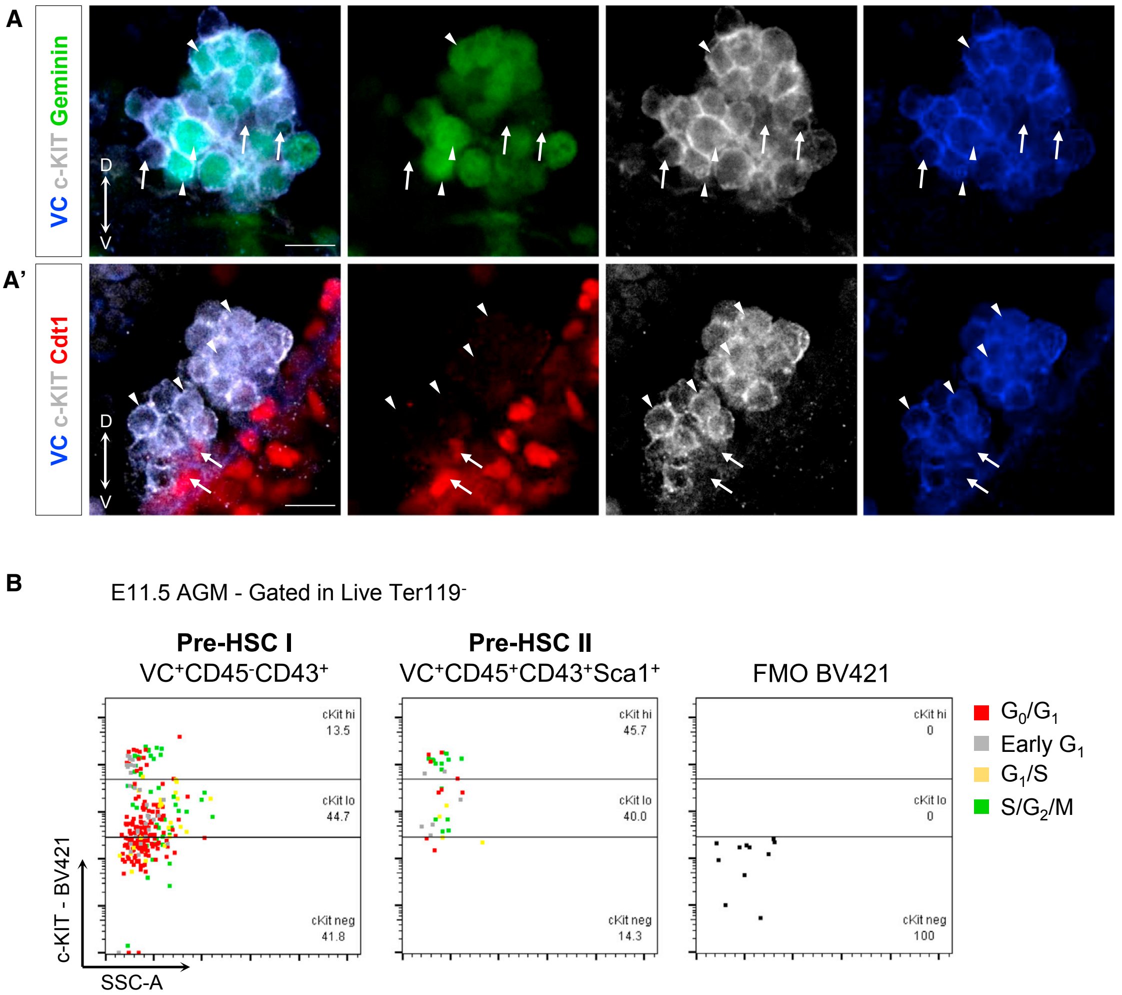


**Figure 4. c-KIT Expression Correlates with Cell-Cycle Status in HSC Precursors**

(A) Localization and Intensity of c-KIT Expression in Intra-aortic Hematopoietic Clusters at E10.5 (A) Geminin-mAG and (A′) Cdt1-mKO2 embryos. White arrowheads show c-KIT$^{hi}$ and Geminin-mAG$^{+}$ (or in A′, Cdt1-mKO2$^{-}$), while white arrows show c-KIT$^{lo}$ and Cdt1-mKO2$^{+}$ (or in A, Geminin-mAG$^{-}$) (at least three embryos).

(B) Representative dot plots of flow cytometry analysis of E11.5 AGM pre-HSCs and correlation of c-KIT expression level with cell-cycle status (three independent experiments).

Scale bar, 10 μm. See also Figures S3 and S4; Table S2.

Pauklin and Vallier, 2013). Proliferation is an important mechanism enabling self-renewal and differentiation of HSCs in the adult (Bowie et al., 2006; Pietras et al., 2011). Here we used Fucci reporter mice to define the proliferative status of the developing HSCs. Our conclusions based on the ratio of Geminin-mAG$^{+}$ and Cdt1-mKO2$^{+}$ cells are consistent with previously described proliferation rates of fetal liver and bone marrow HSCs (Bowie et al., 2006, 2007a; Nygren et al., 2006, 2007b; Takizawa et al., 2011; Fuchs, 2009).

During maturation, the developing HSC pool undergoes massive expansion within the AGM region before colonization of the fetal liver (Rybtsov et al., 2016). HSC maturation occurs through sequential upregulation of hematopoietic markers (CD41, CD43, and CD45) (Taoudi et al., 2008; Rybtsov et al., 2014). Fucci mice enabled visualization and isolation of cells in $G_0/G_1$ (red) and $S/G_2/M$ (green) phases, so that developing HSCs could be studied at both the phenotypic and functional levels (Figure 5A). We found that the Geminin-mAG$^{-}$ but not Geminin-mAG$^{+}$ fraction of the E9.5 pro-HSC population was able to mature into dHSCs, which could reconstitute adult irradiated recipients, indicating that pro-HSCs are not cycling or slowly cycling. By the next day (E10.5), upregulation of CD43 marks the emergence of pre-HSCs type I, which are actively proliferating since both Geminin-mAG$^{+}$ and

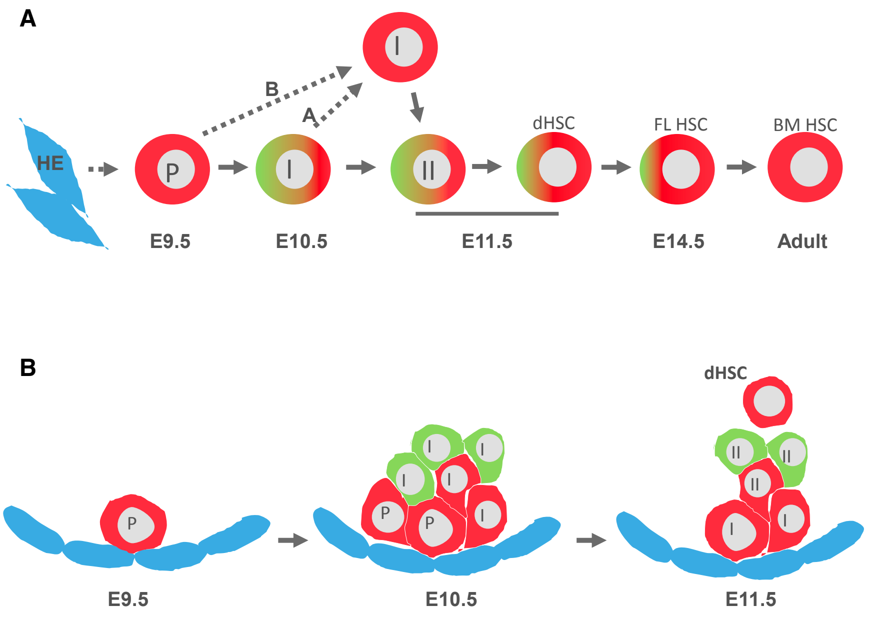


**Figure 5. Model: Changes and Heterogeneity in Proliferative Status of the Developing HSC Lineage and Their Organization within the Intra-aortic Clusters**

(A) Analysis of Fucci reporter mice defined changes in the proliferative status of the HSC precursors during development. The dramatic expansion of the pre-HSC pool during E10.5 correlates with their active proliferation. There might be two scenarios to explain the appearance of slowly cycling pre-HSC type I in E11.5 AGM region; in scenario A a fraction of proliferative E10.5 pre-HSCs type I slows down their cycling and persists as type I until E11.5; in scenario B this is a retarded E9.5 pro-HSC fraction which matured into pre-HSC type I while maintaining slow cycling. Also, dHSCs are slowly cycling like their fetal liver counterparts, while the adult bone marrow HSCs are quiescent.

(B) Slowly cycling cells are frequently found at the base of intra-aortic clusters, while more rapidly cycling cells are located at more apical positions. The proliferative organization of intra-aortic clusters is maintained during development of HSCs in the AGM region and is linked with their maturation. FL, fetal liver; BM, bone marrow; P, pro-HSC; I, pre-HSC type I; II, pre-HSC type II.

Geminin-mAG$^{-}$ fractions can produce dHSCs. Thus, proliferation likely underlies the previously described dramatic expansion of the pre-HSC pool (from 5 cells at early E10 to 50 cells by late E10.5) (Rybtsov et al., 2016). At E11.5 pre-HSCs type II mature and continue to proliferate, with some bias toward the Geminin-mAG$^{-}$ fraction, indicating a slowing down in this process, which becomes apparent in dHSCs. This slowing down of the cell cycle continues further in fetal liver HSCs and, finally, in mainly quiescent bone marrow HSCs (Figure 5A) (Bowie et al., 2007a; Yo et al., 2015).

This study confirms our previous observations that certain states in HSC development can persist for longer than one developmental day (Rybtsov et al., 2016). However, while the proliferative status of E9.5 and E10.5 pro-HSCs is similar, pre-HSCs type I at E11.5 differ from E10.5 by their slow cycling. The origin of the slowly cycling E11.5 pre-HSC type I population is not clear. One possible scenario (Figure 5A, scenario A) is that a fraction of proliferative E10.5 pre-HSCs type I slows down their cycling and persists as type I until E11.5. Another scenario (Figure 5A, scenario B) is that a fraction of retarded E9.5 pro-HSCs matured into pre-HSC type I while maintaining their slow cycling. Whether these distinct pre-HSC fractions can contribute to the heterogeneity of the adult HSC pool (Sieburg et al., 2010; Dykstra et al., 2007; Benz et al., 2012; Ema et al., 2014) needs further investigation. The $G_0/G_1$ status of E11.5 pre-HSC type I observed here contradicts a recent study reporting that these cells are predominantly in $S/G_2/M$ phases, which could be explained by their short-term monitoring of recipients that revealed committed progenitors rather than HSCs (Zhou et al., 2016).

The emergence of HSCs in the AGM region is manifested morphologically by the formation of intra-aortic hematopoietic clusters (Rybtsov et al., 2011, 2014; Taoudi et al., 2008; Yokomizo and Dzierzak, 2010; Kissa and Herbomel, 2010; Boisset et al., 2011). Although this process has not been investigated in detail experimentally, it could be assumed that endothelial-derived HSC precursors proliferate and mature to form the cluster. Our current functional analysis using Fucci mice allowed us to better define the identity of developing HSCs and dynamically map their location within intra-aortic clusters in a stage-specific manner. The day before clusters are formed, at E9.5, single slowly cycling cells of the VC$^{+}$RUNX1$^{+}$CD41$^{+}$ phenotype were found attached to the aortic endothelium. However, since levels of CD41 expression detected under the microscope and by flow cytometry cannot be directly correlated, given the presence of few pro-HSCs per embryo (Rybtsov et al., 2016), this raises the possibility that true, CD41$^{low}$ pro-HSCs escaped our analysis. By the next day when active formation of intra-aortic clusters occurs, slowly cycling ($G_0/G_1$) pro-HSCs remain associated with the endothelium at the base of the cluster, whereas actively cycling ($S/G_2/M$) pre-HSCs type I emerge more apically (Figure 5B). By E11.5, the base of clusters still consists of slowly cycling cells, but

these are now more mature pre-HSC type I. Again, more actively cycling pre-HSC type II develop in more apical positions. This organization was observed in at least 50% of intra-aortic clusters in E10.5 and E11.5 embryos. Similarly structured, although often significantly larger, hematopoietic cell clusters were also observed in extra-embryonic arteries. This suggests that clusters are initiated by slowly cycling precursors, which give rise to more mature actively proliferating precursors moving toward apical positions. This organization of clusters is maintained throughout their maturation, suggesting their growth through predominant expansion of more mature pre-HSCs. This maturation of clusters correlates with progressive quantitative expansion of the pre-HSC population identified functionally by transplantations (Rybtsov et al., 2016).

c-KIT/SCF signaling is essential for HSC biology (Ikuta and Weissman, 1992; Thorén et al., 2008; Ding et al., 2012; Marcelo et al., 2013). We have shown that SCF is ventrally polarized in the AGM region and is a key regulator of stepwise pro-/pre-HSC transitions (Souilhol et al., 2016). c-KIT is expressed in pro-/pre- and dHSCs, and is a principal marker for HSCs in the adult animal (Rybtsov et al., 2014; Kiel et al., 2005). Since c-KIT/SCF signaling is implicated in the regulation of proliferation (Sasaki et al., 2010; Ema et al., 2000; Bashamboo et al., 2006), we studied the organization of c-KIT expressing cells in developing intra-aortic clusters and found that slowly cycling cells, including those at the base of the cluster, express low levels of c-KIT, whereas actively cycling cells express high levels of c-KIT. Although further experimentation is needed to understand this observation mechanistically, this suggests that the stratified proliferative architecture of the cluster is at least partly defined by c-KIT/SCF signaling. Our analysis provides a basis for investigation of cellular and molecular events in maturing intra-aortic clusters in connection with pro/pre-HSC expansion in the AGM region.

Cell proliferation plays an important role in various differentiation processes and needs to be tightly regulated. For example, lengthening $G_1$ phase increases differentiation of neural stem cells into neurons (Lange and Calegari, 2010; Lange et al., 2009); cyclin D in human embryonic stem cells controls balance between neuroectoderm and endoderm specification (Pauklin and Vallier, 2013); and deletion of p27 cell-cycle inhibitor prevents specification of hematopoietic cells from the yolk sac endothelium (Marcelo et al., 2013). The temporal kinetics of pre-HSC proliferation is well controlled: although mature fetal liver HSCs expand, their proliferative activity decreases compared with the AGM region and subsequently, in the adult bone marrow, switches into quiescence associated with low c-KIT expression, which is necessary to prevent exhaustion of the HSC pool (Thorén et al., 2008; Matsuoka et al., 2011; Shin et al., 2014). Our data indicate that pre-HSC expansion within the AGM region is driven by proliferation. It needs to be elucidated in future whether stage-specific proliferative changes per se play a role in HSC maturation.

In summary, our analysis defines changes in proliferative status of the developing HSC lineage at pre-liver stages. We found that dramatic expansion of maturing HSCs correlates with their active proliferation, likely driven by c-KIT/SCF signaling. Proliferative analysis revealed previously concealed heterogeneity within the pre-HSC populations. We describe the proliferative organization of intra-aortic clusters that correlates with the functionally defined status of HSC precursors. This study lays a foundation for molecular analysis of mechanisms underlying HSC development within intra-aortic hematopoietic clusters.

## EXPERIMENTAL PROCEDURES

### Mice

Mice were housed and bred in animal facilities at the University of Edinburgh in compliance with UK Home Office Regulations. Embryos for experiments were obtained from intercrossing heterozygous hCdt1(30/120)-mKO2 (#610) and hGeminin(1/110)-mAG (#474) mice (Sakaue-Sawano et al., 2008; Yo et al., 2015; Zielke and Edgar, 2015) or from C57BL/6 CD45.2/2 mice. The day of discovery of the vaginal plug was designated as day 0.5. The embryos were additionally staged based on somite pair (sp) numbers (E9.5 = 26–29 sp, E10.5 = 30–38 sp, E11.5 = 41–45 sp). C57BL/6 CD45.1/2 mice were used as transplant recipients and C57BL/6 CD45.1/1 as a source of carrier cells. All experiments with animals were approved under a Project License granted by the Home Office (UK) and the University of Edinburgh Ethical Review Committee, and conducted in accordance with local guidelines.

### Flow Cytometry and Cell Sorting

Single-cell suspensions from the AGM region or fetal liver were prepared by dispase/collagenase-mediated dissociation, while single-cell suspensions from bone marrow were obtained by flushing the tibias and femurs with a 26-gauge syringe needle (BD Microlance). Antibodies used for staining of cells were: anti-CD45-BV450 or BV650 (BD Horizon, clone 30F11), anti-VE-cadherin-A647 (BioLegend, Clone eBioBV13), biotinylated anti-VE-cadherin (clone 11.D4.1) followed by incubation with streptavidin-APC (BD Pharmingen), anti-CD43-PE or biotinylated anti-CD43 (eBioscience, clone eBioR2/60) followed by incubation with streptavidin-BV650 (BioLegend), anti-CD41-PE or BV421 (BioLegend, clone MWReg30), anti-Sca1-BV421 or PE-Cy7 (eBioscience, clone D7), anti-c-KIT/CD117-BV421 (BioLegend, clone 2B8), anti-CD150-APC (BioLegend, clone TC15-12F12.2), anti-CD48-PerCPefluor710 (BioLegend, clone HM48-1), anti-Ki67-AF647 (BD Pharmingen, clone B56), DAPI (Biotium), anti-Ter119-PerCp-Cy5.5 or biotinylated anti-Ter119 (eBioscience), biotinylated anti-B220/CD45R (eBioscience, clone RA3-6B2), biotinylated anti-CD3e (eBioscience, clone 145-2C11), and biotinylated anti-Gr1 (eBioscience, clone RB6-8C5). Lineage depletion of bone marrow

and fetal liver samples was performed by streptavidin particles (BD IMag) according to the manufacturer's instructions. 7-Aminoactinomycin D viability staining solution, live-dead dye Zombie Aqua (BioLegend), or Infra-Red (Invitrogen) were used to exclude dead cells and gates were set using appropriate fluorescence minus one (FMO) controls. Flow-cytometry analysis was performed on a Fortessa LSR using FACSDiva software, while analysis was done using FlowJo 10. Sorting was performed on a FACSAriaII using FACSDiva software. Correlation analysis between c-KIT levels and cell-cycle phases was performed using GraphPad Prism. The different cell-cycle fractions were plotted against c-KIT in FlowJo and segmented in equal parts across the c-KIT axis. The cell numbers within each segment/gate were extracted from FlowJo and used for the correlation analysis. A correlation coefficient (r) of +1 indicates perfect positive correlation (i.e., when x increases then y increases), whereas −1 shows negative/inverse correlation (i.e., when x increases then y decreases). A correlation coefficient of 0 shows that the two variables do not vary at all. $R^2$, or coefficient of determination, is the fraction of the variance in the two variables that is shared. The p values in this analysis show whether the correlation is due to random sampling. Nonlinear regression analysis was used to draw a graph with a smooth curve that fits the data.

## OP9 Co-aggregates

E9.5 caudal parts or E10.5–E11.5 AGM regions were isolated and for some experiments were subdissected into AoV, AoD, and UGRs. The notochord was included in the AoD. Sorted populations were co-aggregated with OP9 stromal cells as previously described (Rybtsov et al., 2011, 2014). In all experiments, 1 embryo equivalent (ee), defined as a unit of cells equivalent to the number present in one organ (e.g., 0.2ee corresponds to 20% of cells present in one AGM region), of sorted cells was co-aggregated with 100,000 OP9 cells. Cell aggregates were cultured at the liquid-gas interface on 0.8 μm of mixed cellulose MF-membranes (AAWP02500, Millipore) for 5–7 days (37°C, 5% $CO_2$) in 5 mL of Iscove's modified Dulbecco's medium (IMDM, Invitrogen), 20% fetal calf serum (FCS; HyClone, ThermoScientific) supplemented with L-glutamine (4 mM), penicillin/streptomycin (50 units/mL) and 100 ng/mL SCF, 100 ng/ml IL-3, and 100 ng/mL Flt3l (all purchased from Peprotech). Suitable batches of FCS supporting effective maturation of pre-HSCs were selected after pre-testing in preliminary transplantation experiments (Taoudi et al., 2008). Cells from E9.5–E10.5 AGM regions were cultured for 7 days, while cells from E11.5 AGM regions were cultured for 5 days.

## HSC Transplantation and CFU-C Assay

AGM tissues from C57BL/6 CD45.2/2 embryos were pooled and cell suspensions obtained after dissociation with collagenase/dispase (Roche) for 40 min at 37°C. Dissociated cells were plated in methylcellulose culture that contains cytokines (MethoCult3434 medium; STEMCELL Technologies) according to the manufacturer's instructions. Donor cells were injected intravenously into C57BL/6 CD45.1/2 sublethally irradiated (1,150 rad) mice along with 50,000 C57BL/6 CD45.1/1 bone marrow carrier cells. The amount of transplanted cells is expressed in ee and the dose injected for each experiment was chosen based on the expected outcome of dHSC numbers, which can vary depending on the developmental stage.

Long-term hematopoietic repopulation by donor cells was assessed in peripheral blood between 14 and 16 weeks after transplantation. Peripheral blood was collected by bleeding the tail vein into 500 μL of 5 mM EDTA/PBS. Erythrocytes were depleted using PharM Lyse (BD Biosciences). Cells were stained with anti-CD16/32 (Fc-block), anti-CD45.1-V450 (cloneA20), and anti-CD45.2-APC (clone 104) monoclonal antibodies (eBioscience) and analyzed using a FACSCalibur. Data were analyzed in FlowJo software (TreeStar). Mice exhibiting >5% of donor chimerism were considered to be repopulated with dHSCs. Different groups of repopulated mice were compared using one-way ANOVA or t test (*p < 0.05, **p < 0.01, ***p < 0.005, ****p < 0.0001). Limiting dilution analysis of fetal liver was performed using ELDA software (Hu and Smyth, 2009).

## Immunofluorescence

Whole-mount immunostaining was performed as previously described (Yokomizo et al., 2012) with slight modifications. Embryos dissected from the yolk sac and amnion were fixed with 2% paraformaldehyde or cold acetone and, following dehydration by increasing concentrations of methanol, the head, limbs, and one body wall were removed. After rehydration in 50% methanol, washing with PBS, and blocking in 50% FCS/0.5% Triton X-100, the samples were incubated overnight with antibodies. For staining with antibodies from the same species, incubations were performed sequentially. Primary antibodies used were unconjugated goat anti-mouse CD43 (Santa Cruz Biotechnology, clone M19), rat anti-mouse VE-cadherin (BD Pharmingen, clone 11D4.1), rat anti-mouse CD45 (BD Pharmingen, clone 30-F11), rabbit anti-mouse RUNX1 (Abcam, clone EPR3099), rat anti-mouse CD41 (BD Pharmingen, clone MWReg30), rat anti-mouse c-KIT (BioLegend, clone 2B8), rabbit anti-mouse Ki67 (Abcam, clone SP6), rabbit anti-mAG (MBL International), and rabbit anti-mKO2 (MBL International), and these were detected by the secondary antibodies anti-goat NL557 (R&D Systems), anti-rat Alexa Fluor 647 (Invitrogen), or anti-rat Alexa 488 (Invitrogen) and anti-rabbit Alexa Fluor 647 (Abcam). After washing, the embryos were dehydrated with methanol and cleared with BABB (one part benzyl alcohol, two parts benzyl benzoate) solution (Yokomizo and Dzierzak, 2010). Secondary antibody only controls were used in all the experiments. Imaging of live sections was performed as previously described (Boisset et al., 2011) with slight modifications. E10.5 mouse embryos were dissected and cut into slices of 300-μm thickness using a tissue chopper. Slices were stained against c-KIT-APC (eBioscience, clone 2B8) for 10 min at 4°C, then placed in glass-bottom dishes (MatTek) and covered with low-melting-point agarose gel (4%). The gel was then covered with IMDM without phenol red. Images were acquired with an inverted confocal microscope (Leica SP8) and processed using Volocity software.

# SUPPLEMENTAL INFORMATION

Supplemental Information includes four figures and two tables and can be found with this article online at http://dx.doi.org/10.1016/j.stemcr.2017.04.003.

## AUTHOR CONTRIBUTIONS

A.B., S.R., C.S., A.B.-C., D.H., and S.Z. performed experiments. A.B. and A.M. designed the research. A.B. analyzed data and made figures. A.B. and A.M. wrote the paper. P.T. provided important advice and support.

## ACKNOWLEDGMENTS

The authors thank J. Verth, C. Manson, J. Agnew, and R. McInnis for assistance with mouse maintenance and breeding; C. Watt., C. Flockhart, C. Forrest, A. Dyer for irradiations; O. Rodriguez, F. Rossi, and C. Cryer for cell sorting; and V. Berno and B. Vernay for help with microscopy. We thank A. Sakaue-Sawano for providing Fucci reporter mice. We thank S. Zuyev for helpful discussions on the correlation analysis. We thank S. Gordon-Keylock, A. McGarvey, J. Easterbrook, E. Crosse, and S. Heinrichs for helpful comments. This work was supported by Bloodwise (former LLR), BBSRC, MRC, and the Wellcome Trust.

Received: December 21, 2016
Revised: April 3, 2017
Accepted: April 4, 2017
Published: May 4, 2017

## REFERENCES

Bashamboo, A., Taylor, A.H., Samuel, K., Panthier, J.J., Whetton, A.D., and Forrester, L.M. (2006). The survival of differentiating embryonic stem cells is dependent on the SCF-KIT pathway. J. Cell Sci. *119* (Pt 15), 3039–3046.

Benz, C., Copley, M.R., Kent, D.G., Wohrer, S., Cortes, A., Aghaeepour, N., Ma, E., Mader, H., Rowe, K., Day, C., et al. (2012). Hematopoietic stem cell subtypes expand differentially during development and display distinct lymphopoietic programs. Cell Stem Cell *10*, 273–283.

Boisset, J.C., Andrieu-Soler, C., van Cappellen, W.A., Clapes, T., and Robin, C. (2011). Ex vivo time-lapse confocal imaging of the mouse embryo aorta. Nat. Protoc. *6*, 1792–1805.

Boisset, J.C., Clapes, T., Klaus, A., Papazian, N., Onderwater, J., Mommaas-Kienhuis, M., Cupedo, T., and Robin, C. (2015). Progressive maturation toward hematopoietic stem cells in the mouse embryo aorta. Blood *125*, 465–470.

Bowie, M.B., McKnight, K.D., Kent, D.G., McCaffrey, L., Hoodless, P.A., and Eaves, C.J. (2006). Hematopoietic stem cells proliferate until after birth and show a reversible phase-specific engraftment defect. J. Clin. Invest. *116*, 2808–2816.

Bowie, M.B., Kent, D.G., Dykstra, B., McKnight, K.D., McCaffrey, L., Hoodless, P.A., and Eaves, C.J. (2007a). Identification of a new intrinsically timed developmental checkpoint that reprograms key hematopoietic stem cell properties. Proc. Natl. Acad. Sci. USA *104*, 5878–5882.

Bowie, M.B., Kent, D.G., Dykstra, B., McKnight, K.D., McCaffrey, L., Hoodless, P.A., and Eaves, C.J. (2007b). Steel factor responsiveness regulates the high self-renewal phenotype of fetal hematopoietic stem cells. Blood *109*, 5043–5048.

Budirahardja, Y., and Gönczy, P. (2009). Coupling the cell cycle to development. Development *136*, 2861–2872.

Ciau-Uitz, A., Patient, R., and Medvinsky, A. (2016). Ontogeny of the hematopoietic system. In Encyclopedia of Immunobiology, M.J.H. Ratcliffe, ed. (Elsevier), pp. 1–14.

Ding, L., Saunders, T.L., Enikolopov, G., and Morrison, S.J. (2012). Endothelial and perivascular cells maintain haematopoietic stem cells. Nature *481*, 457–462.

Dykstra, B., Kent, D., Bowie, M., McCaffrey, L., Hamilton, M., Lyons, K., Lee, S.J., Brinkman, R., and Eaves, C. (2007). Long-term propagation of distinct hematopoietic differentiation programs in vivo. Cell Stem Cell *1*, 218–229.

Ema, H., and Nakauchi, H. (2000). Expansion of hematopoietic stem cells in the developing liver of a mouse embryo. Blood *95*, 2284–2288.

Ema, H., Takano, H., Sudo, K., and Nakauchi, H. (2000). In vitro self-renewal division of hematopoietic stem cells. J. Exp. Med. *192*, 1281–1288.

Ema, H., Morita, Y., and Suda, T. (2014). Heterogeneity and hierarchy of hematopoietic stem cells. Exp. Hematol. *42*, 74–82.

Fuchs, E. (2009). The tortoise and the hair: slow-cycling cells in the stem cell race. Cell *137*, 811–819.

Garcia-Porrero, J., Godin, I., and Dieterlen-Lièvre, F. (1995). Potential intraembryonic hemogenic sites at pre-liver stages in the mouse. Anat. Embryol. (Berl) *192*, 423–435.

Gordon-Keylock, S., Sobiesiak, M., Rybtsov, S., Moore, K., and Medvinsky, A. (2013). Mouse extraembryonic arterial vessels harbor precursors capable of maturing into definitive HSCs. Blood *122*, 2338–2345.

Hu, Y., and Smyth, G.K. (2009). ELDA: extreme limiting dilution analysis for comparing depleted and enriched populations in stem cell and other assays. J. Immunol. Methods *347*, 70–78.

Ikuta, K., and Weissman, I.L. (1992). Evidence that hematopoietic stem cells express mouse c-kit but do not depend on steel factor for their generation. Proc. Natl. Acad. Sci. USA *89*, 1502–1506.

Jude, C.D., Gaudet, J.J., Speck, N.A., and Ernst, P. (2008). Leukemia and hematopoietic stem cells: balancing proliferation and quiescence. Cell Cycle *7*, 586–591.

Kaldis, P., and Richardson, H.E. (2012). When cell cycle meets development. Development *139*, 225–230.

Kiel, M.J., Yilmaz, O.H., Iwashita, T., Yilmaz, O.H., Terhorst, C., and Morrison, S.J. (2005). SLAM family receptors distinguish hematopoietic stem and progenitor cells and reveal endothelial niches for stem cells. Cell *121*, 1109–1121.

Kissa, K., and Herbomel, P. (2010). Blood stem cells emerge from aortic endothelium by a novel type of cell transition. Nature *464*, 112–115.

Kumaravelu, P., Hook, L., Morrison, A.M., Ure, J., Zhao, S., Zuyev, S., Ansell, J., Medvinsky, A., et al. (2002). Quantitative developmental anatomy of definitive haematopoietic stem cells/long-term repopulating units (HSC/RUs): role of the aorta-gonad-mesonephros (AGM) region and the yolk sac in colonisation of the mouse embryonic liver. Development *129*, 4891–4899.

Lange, C., and Calegari, F. (2010). Cdks and cyclins link G1 length and differentiation of embryonic, neural and hematopoietic stem cells. Cell Cycle *9*, 1893–1900.

Lange, C., Huttner, W.B., and Calegari, F. (2009). Cdk4/cyclinD1 overexpression in neural stem cells shortens G1, delays neurogenesis, and promotes the generation and expansion of basal progenitors. Cell Stem Cell *5*, 320–331.

Liakhovitskaia, A., Rybtsov, S., Smith, T., Batsivari, A., Rybtsova, N., Rode, C., de Bruijn, M., Buchholz, F., Gordon-Keylock, S., Zhao, S., and Medvinsky, A. (2014). Runx1 is required for progression of $CD41^+$ embryonic precursors into HSCs but not prior to this. Development *141*, 3319–3323.

Marcelo, K.L., Sills, T.M., Coskun, S., Vasavada, H., Sanglikar, S., Goldie, L.C., and Hirschi, K.K. (2013). Hemogenic endothelial cell specification requires c-Kit, Notch signaling, and p27-mediated cell-cycle control. Dev. Cell *27*, 504–515.

Matsuoka, Y., Sasaki, Y., Nakatsuka, R., Takahashi, M., Iwaki, R., Uemura, Y., and Sonoda, Y. (2011). Low level of C-kit expression marks deeply quiescent murine hematopoietic stem cells. Stem Cells *29*, 1783–1791.

Medvinsky, A., and Dzierzak, E. (1996). Definitive hematopoiesis is autonomously initiated by the AGM region. Cell *86*, 897–906.

Medvinsky, A., Rybtsov, S., and Taoudi, S. (2011). Embryonic origin of the adult hematopoietic system: advances and questions. Development *138*, 1017–1031.

Mendelson, A., and Frenette, P.S. (2014). Hematopoietic stem cell niche maintenance during homeostasis and regeneration. Nat. Med. *20*, 833–846.

Morrison, S.J., and Scadden, D.T. (2014). The bone marrow niche for haematopoietic stem cells. Nature *505*, 327–334.

Müller, A.M., Medvinsky, A., Strouboulis, J., Grosveld, F., and Dzierzak, E. (1994). Development of hematopoietic stem cell activity in the mouse embryo. Immunity *1*, 291–301.

Nakamura-Ishizu, A., Takizawa, H., and Suda, T. (2014). The analysis, roles and regulation of quiescence in hematopoietic stem cells. Development *141*, 4656–4666.

North, T., Gu, T.L., Stacy, T., Wang, Q., Howard, L., Binder, M., Marín-Padilla, M., and Speck, N.A. (1999). Cbfa2 is required for the formation of intra-aortic hematopoietic clusters. Development *126*, 2563–2575.

Nygren, J.M., Bryder, D., and Jacobsen, S.E.W. (2006). Prolonged cell cycle transit is a defining and developmentally conserved hemopoietic stem cell property. J. Immunol. *177*, 201–208.

Parish, C.R. (1999). Fluorescent dyes for lymphocyte migration and proliferation studies. Immunol. Cell Biol. *77*, 499–508.

Passegué, E., Wagers, A.J., Giuriato, S., Anderson, W.C., and Weissman, I.L. (2005). Global analysis of proliferation and cell cycle gene expression in the regulation of hematopoietic stem and progenitor cell fates. J. Exp. Med. *202*, 1599–1611.

Pauklin, S., and Vallier, L. (2013). The cell-cycle state of stem cells determines cell fate propensity. Cell *155*, 135–147.

Pietras, E.M., Warr, M.R., and Passegue, E. (2011). Cell cycle regulation in hematopoietic stem cells. J. Cell Biol. *195*, 709–720.

Rybtsov, S., Sobiesiak, M., Taoudi, S., Souilhol, C., Senserrich, J., Liakhovitskaia, A., Ivanovs, A., Frampton, J., Zhao, S., and Medvinsky, A. (2011). Hierarchical organization and early hematopoietic specification of the developing HSC lineage in the AGM region. J. Exp. Med. *208*, 1305–1315.

Rybtsov, S., Batsivari, A., Bilotkach, K., Paruzina, D., Senserrich, J., Nerushev, O., and Medvinsky, A. (2014). Tracing the origin of the HSC hierarchy reveals an SCF-dependent, IL-3-independent $CD43^-$ embryonic precursor. Stem Cell Rep. *3*, 489–501.

Rybtsov, S., Ivanovs, A., Zhao, S., and Medvinsky, A. (2016). Concealed expansion of immature precursors underpins acute burst of adult HSC activity in foetal liver. Development *143*, 1284–1289.

Sakaue-Sawano, A., Kurokawa, H., Morimura, T., Hanyu, A., Hama, H., Osawa, H., Kashiwagi, S., Fukami, K., Miyata, T., Miyoshi, H., et al. (2008). Visualizing spatiotemporal dynamics of multicellular cell-cycle progression. Cell *132*, 487–498.

Sasaki, T., Mizuochi, C., Horio, Y., Nakao, K., Akashi, K., and Sugiyama, D. (2010). Regulation of hematopoietic cell clusters in the placental niche through SCF/Kit signaling in embryonic mouse. Development *137*, 3941–3952.

Seita, J., and Weissman, I.L. (2010). Hematopoietic stem cell: self-renewal versus differentiation. Wiley Interdiscip. Rev. Syst. Biol. Med. *2*, 640–653.

Shin, J.Y., Hu, W., Naramura, M., and Park, C.Y. (2014). High c-Kit expression identifies hematopoietic stem cells with impaired self-renewal and megakaryocytic bias. J. Exp. Med. *211*, 217–231.

Sieburg, H.B., Cho, R.H., Dykstra, B., Uchida, N., Eaves, C.J., and Muller-Sieburg, C.E. (2010). The hematopoietic stem compartment consists of a limited number of discrete stem cell subsets the hematopoietic stem compartment consists of a limited number of discrete stem cell subsets. Blood *107*, 2311–2316.

Souilhol, C., Gonneau, C., Lendinez, J.G., Batsivari, A., Rybtsov, S., Wilson, H., Morgado-Palacin, L., Hills, D., Taoudi, S., Antonchuk, J., et al. (2016). Inductive interactions mediated by interplay of asymmetric signalling underlie development of adult haematopoietic stem cells. Nat. Commun. *7*, 10784.

Swiers, G., Baumann, C., O'Rourke, J., Giannoulatou, E., Taylor, S., Joshi, A., Moignard, V., Pina, C., Bee, T., Kokkaliaris, K.D., et al. (2013). Early dynamic fate changes in haemogenic endothelium characterized at the single-cell level. Nat. Commun. *4*, 2924.

Takizawa, H., Regoes, R.R., Boddupalli, C.S., Bonhoeffer, S., and Manz, M.G. (2011). Dynamic variation in cycling of hematopoietic stem cells in steady state and inflammation. J. Exp. Med. *208*, 273–284.

Taoudi, S., and Medvinsky, A. (2007). Functional identification of the hematopoietic stem cell niche in the ventral domain of the embryonic dorsal aorta. Proc. Natl. Acad. Sci. USA *104*, 9399–9403.

Taoudi, S., Gonneau, C., Moore, K., Sheridan, J.M., Blackburn, C.C., Taylor, E., and Medvinsky, A. (2008). Extensive hematopoietic stem cell generation in the AGM region via maturation of $VE\text{-}Cadherin^+$ $CD45^+$ pre-definitive HSCs. Cell Stem Cell *3*, 99–108.

Thorén, L., Liuba, K., Bryder, D., Nygren, J.M., Jensen, C.T., Qian, H., Antonchuk, J., and Jacobsen, S.E. (2008). Kit regulates

maintenance of quiescent hematopoietic stem cells. J. Immunol. *180*, 2045–2053.

Wilson, A., Laurenti, E., Oser, G., van der Wath, R.C., Blanco-Bose, W., Jaworski, M., Offner, S., Dunant, C.F., Eshkind, L., Bockamp, E., et al. (2008). Hematopoietic stem cells reversibly switch from dormancy to self-renewal during homeostasis and repair. Cell *135*, 1118–1129.

Yo, M., Sakaue-Sawano, A., Noda, S., Miyawaki, A., and Miyoshi, H. (2015). Fucci-guided purification of hematopoietic stem cells with high repopulating activity. Biochem. Biophysical Res. Commun. *457*, 7–11.

Yoder, M.C., Hiatt, K., Dutt, P., Mukherjee, P., Bodine, D.M., and Orlic, D. (1997). Characterization of definitive lymphohematopoietic stem cells in the day 9 murine yolk sac. Immunity *7*, 335–344.

Yokomizo, T., Yamada-Inagawa, T., Yzaguirre, A.D., Chen, M.J., Speck, N.A., and Dzierzak, E. (2012). Whole-mount three-dimensional imaging of internally localized immunostained cells within mouse embryos. Nat. Protoc. *7*, 421–431.

Yokomizo, T., and Dzierzak, E. (2010). Three-dimensional cartography of hematopoietic clusters in the vasculature of whole mouse embryos. Development *137*, 3651–3661.

Zhou, F., Li, X., Wang, W., Zhu, P., Zhou, J., He, W., Ding, M., Xiong, F., Zheng, X., Li, Z., et al. (2016). Tracing haematopoietic stem cell formation at single-cell resolution. Nature *533*, 1–17.

Zielke, N., and Edgar, B.A. (2015). FUCCI sensors: powerful new tools for analysis of cell proliferation. Wiley Interdiscip. Rev. Dev. Biol. *4*, 469–487.

Zovein, A.C., Turlo, K.A., Ponec, R.M., Lynch, M.R., Chen, K.C., Hofmann, J.J., Cox, T.C., Gasson, J.C., and Iruela-Arispe, M.L. (2010). Vascular remodeling of the vitelline artery initiates extravascular emergence of hematopoietic clusters. Blood *116*, 3435–3444.

**Stem Cell Reports, Volume *8***

## Supplemental Information

# Understanding Hematopoietic Stem Cell Development through Functional Correlation of Their Proliferative Status with the Intra-aortic Cluster Architecture

**Antoniana Batsivari, Stanislav Rybtsov, Celine Souilhol, Anahi Binagui-Casas, David Hills, Suling Zhao, Paul Travers, and Alexander Medvinsky**

**Supplementary Figure 1**. Related to Figure 1.

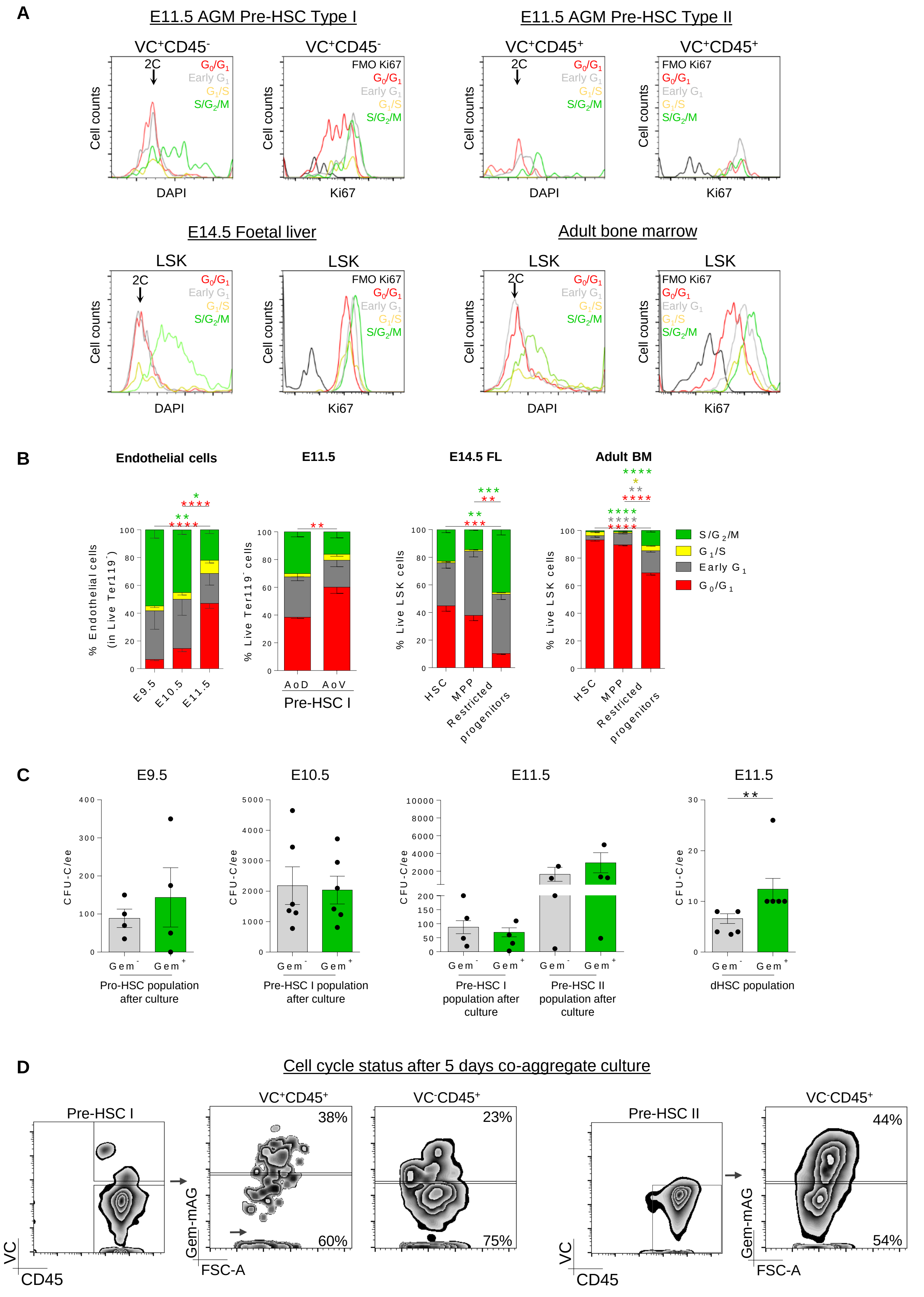

**Supplementary Figure 1**. Related to Fig. 1. **Validation of Fucci reporters and cell cycle analysis of haematopoietic populations.**

(A) Validation of the Fucci reporters (Geminin-mAG and Cdt1-mKO2) in haematopoietic populations at various stages with Ki67 and DAPI staining (2C, diploid DNA) (2-3 independent experiments). All populations are gated on Live Lineage$^-$ cells. (B) Flow cytometry of various populations of Fucci embryos and adults (3-4 independent experiments). The erro r bars show SEM. The endothelial population is identified as VC$^+$CD45$^-$CD41$^-$CD43$^-$ and the pre-HSC Type I population as VC$^+$CD45$^-$CD41$^-$CD43$^+$, in the AGM region. The HSC population is identified as LSK CD150$^+$CD48$^-$, the MPP as LSK CD150$^-$CD48$^-$ and the restricted progenitors as LSK CD150$^-$CD48$^+$, both in FL and BM. (C) In vitro methylcellulose assay of pro/pre-HSC sorted populations after co-aggregate culture and dHSC populations directly after sorting (at least 4 independent experiments). The error bars show SEM . (D) Representative flow cytometry plots of E11.5 pre-HSC I and II sorted populations after 5 days co-aggregate culture (at least 3 independent experiments). Geminin-mAG$^{-/+}$ fractions had no difference in their output in terms of cell cycle status. AoV, ventral part of dorsal aorta,; AoD, Dorsal part of dorsal aorta; FL, foetal liver; BM, bone marrow. **$p<0.00$ 5, ***$p<0.0005$, ****$p<0.0001$ .

## **Supplementary Figure 2**. Related to Figures 2 and 3

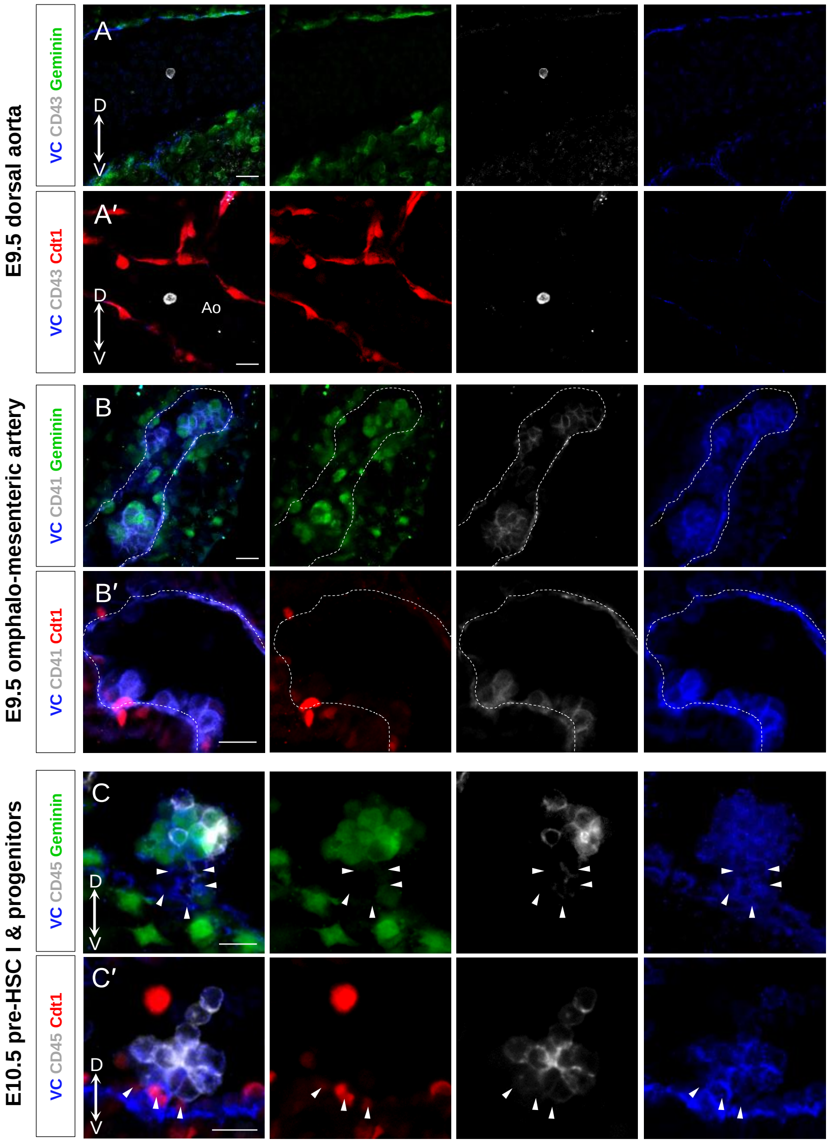


**Supplementary Figure 2**. Related to Fig. 2, 3. **Cell cycle status of E9.5 and E10.5 HSC precursors.**

(A), (B), (C) Geminin-mAG and (A′), (B′), (C′) Cdt1-mKO2 embryos stained for different haematopoietic markers. (A), (A′) CD43 is not expressed in the dorsal aorta of E9.5 embryos. (B), (B′) CD41 is expressed in the E9.5 OMA cell clusters, which are Geminin-mAG$^{+}$Cdt1-mKO2$^{-}$. (C), (C′) CD45 is localised at the top of the E10.5 intra-aortic clusters and marks mainly progenitors that are actively cycling (Geminin-mAG$^{+}$Cdt1-mKO2$^{-}$). White arrowheads show the $G_0/G_1$ (Geminin-mAG$^{-}$Cdt1-mKO2$^{+}$) cells that CD45$^{-}$. Scale bar, 15 µm.

# **Supplementary Figure 3.** Related to Figure 3.

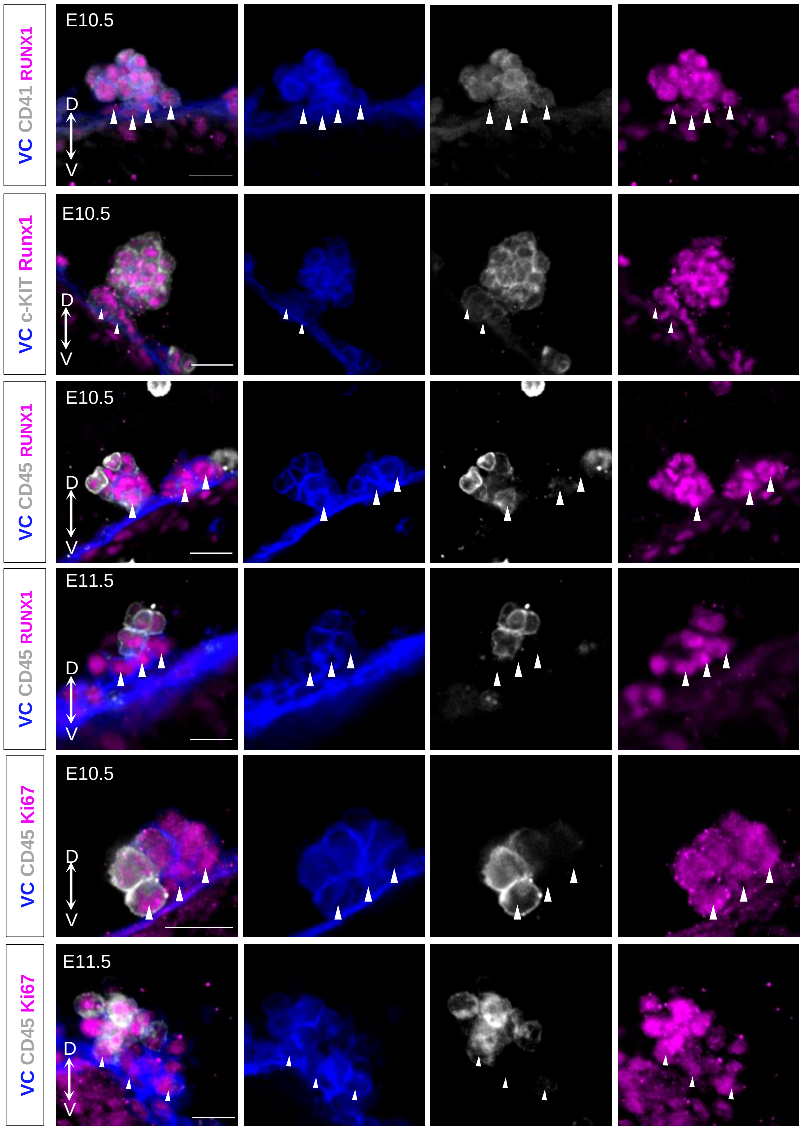


**Supplementary Figure 3.** Related to Fig. 3 & 4. **Characterisation of intra-aortic clusters with various haematopoietic markers.**

Representative images of wild type E10.5-11.5 embryos showing the expression pattern of RUNX1, CD41, c-KIT, CD45 and Ki67 within intra-aortic clusters. Of note, the cells at the base of these clusters are haematopoietic (white arrowheads). Scale bar, 15 µm.

**Supplementary Figure 4.** Related to Figure 3 and 4.

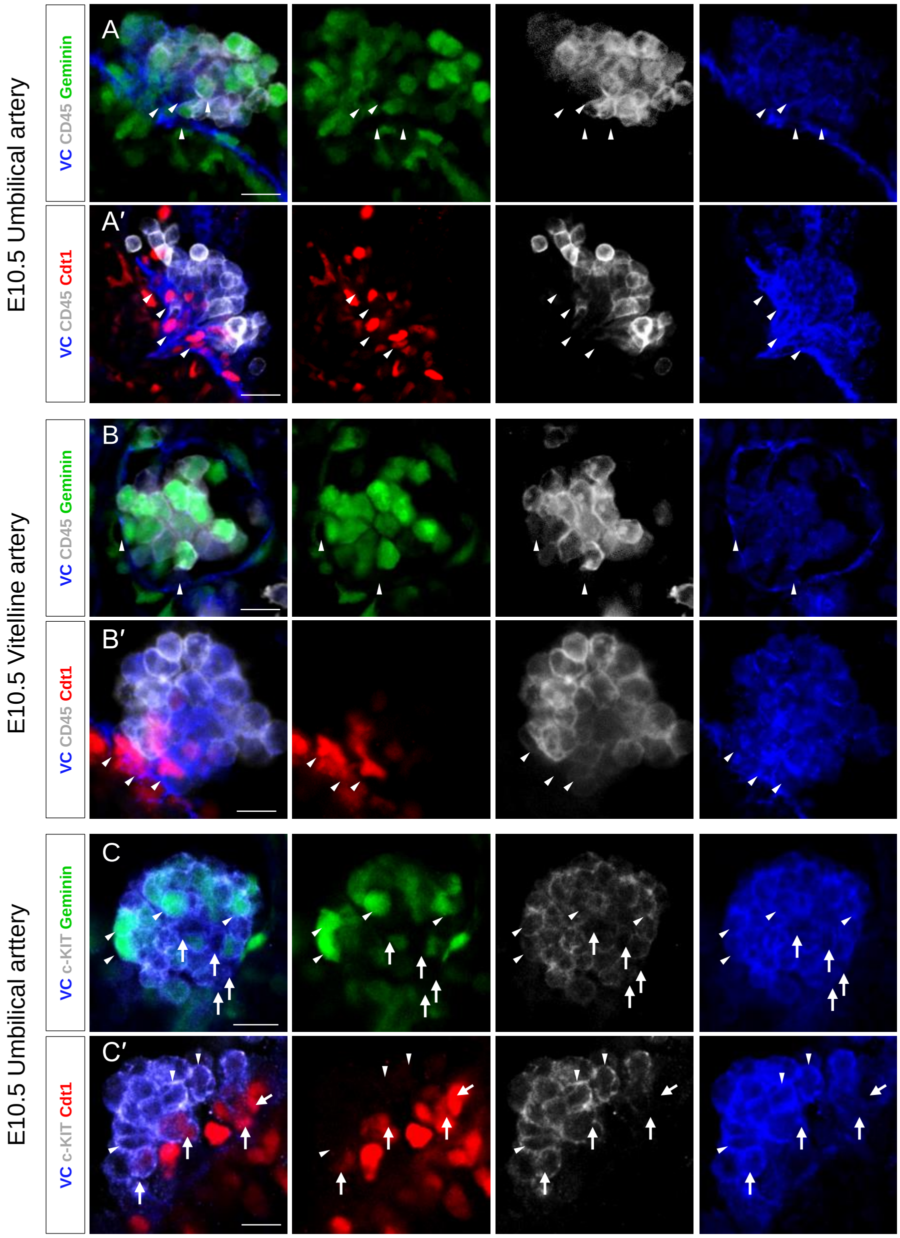


**Supplementary Figure 4.** Related to Fig. 3 & 4. **Cell cycle status of haematopoietic clusters in the extra-embryonic vessels.** (A), (B), (C) Geminin-mAG and (A′), (B′), (C′) Cdt1-mKO2 embryos stained for different haematopoietic markers. (A), (A′), (B), (B′) Representative images of haematopoietic clusters with slowly cycling cells (Geminin-mAG$^{-}$Cdt1-mKO2$^{+}$) at their base in umbilical and vitelline arteries (white arrowheads) in E10.5 embryos. (C), (C′) c-KIT expression in umbilical artery clusters showing that c-KIT$^{lo}$ cells are Geminin-mAG$^{-}$Cdt1-mKO2$^{+}$ (white arrows), while c-KIT$^{hi}$ expressing cells are Geminin-mAG$^{+}$Cdt1-mKO2$^{-}$ (white arrowheads). Scale bar, 10µm.

**Supplementary Table 1.** Related to Figure 1.

A

| Experiment | No. cells/ee (1 aggregate) | | No. cells/aggregate after culture | | Dose transplanted/ recipient |
|---|---|---|---|---|---|
| | Gem-mAG$^-$ | Gem-mAG$^+$ | Gem-mAG$^-$ | Gem-mAG$^+$ | |
| E9.5 pro-HSC Expt.1 | 130 | 36 | 66612 | 41244 | 4 ee |
| E9.5 pro-HSC Expt.2 | 32 | 20 | 65743 | 43956 | 5 ee |
| E10.5 pre-HSC I Expt.1 | 117 | 62 | 1231984 | 1060184 | 4 ee |
| E10.5 pre-HSC I Expt.2 | 131 | 56 | 934617 | 1226718 | 4 ee |
| E10.5 pre-HSC I Expt.3 | 181 | 91 | 1513570 | 892790 | 4 ee |
| E11.5 pre-HSC I Expt.1 | 1209 | 1041 | 43850 | 65710 | 1 ee |
| E11.5 pre-HSC I Expt.2 | 1475 | 960 | 39840 | 33010 | 1 ee |
| E11.5 pre-HSC I Expt.3 | 1381 | 314 | 29050 | 23710 | 1 ee |
| E11.5 pre-HSC I Expt.4 | 1346 | 935 | 48213 | 37854 | 1 ee |
| E11.5 pre-HSC I Expt.5 | 1399 | 521 | 26084 | 30588 | 1 ee |
| E11.5 pre-HSC II Expt.1 | 397 | 1041 | 69312 | 131835 | 1 ee |
| E11.5 pre-HSC II Expt.2 | 437 | 578 | 70966 | 87188 | 1 ee |
| E11.5 pre-HSC II Expt.3 | 794 | 332 | 94278 | 125761 | 1 ee |
| E11.5 pre-HSC II Expt.4 | 1143 | 771 | 80580 | 93525 | 1 ee |
| E11.5 pre-HSC II Expt.5 | 716 | 411 | 79890 | 89050 | 1 ee |

B

| Experiment | No. cells transplanted/recipient | | | |
|---|---|---|---|---|
| | Gem-mAG$^-$ | | Gem-mAG$^+$ | |
| E11.5 dHSC Expt.1 | 20487 | | 10471 | |
| E11.5 dHSC Expt.2 | 45567 | | 29463 | |
| E11.5 dHSC Expt.3 | 32467 | | 12610 | |
| E11.5 dHSC Expt.4 | 102000 | | 84500 | |
| E11.5 dHSC Expt.5 | 182909 | | 53090 | |
| E11.5 dHSC Expt.6 | 137181 | | 39181 | |
| | $G_0/G_1$ | Early $G_1$ | $G_1/S$ | $S/G_2/M$ |
| E14.5 FL Expt.1 | 342 | 465 | 222 | 414 |
| E14.5 FL Expt.2 | 455 | 426 | 356 | 453 |
| E14.5 FL Expt.3 | 34 | 36 | 5 | 46 |
| Adult BM Expt.1 | 6417 | 1839 | 183 | 1204 |
| Adult BM Expt.2 | 1287 | 116 | 61 | 46 |

**Supplementary Table 1.** Related to Figure 1. **Cell numbers for co-aggregate culture and transplantations.** (A) E9.5, 10.5 and 11.5 Geminin-mAG reporter embryos were sorted for pro/pre-HSCs. Representative numbers of sorted cells used for one co-aggregate (1 ee), representative total cell numbers of one co-aggregate after culture and the dose transplanted in each experiment are shown in the table. (B) The number of sorted cells from E11.5 AGM region (Geminin-mAG reporter embryos), foetal liver and adult bone marrow (Fucci embryos and adults) that were transplanted directly in recipients is shown in this table.

**Supplementary Table 2.** Related to Figure 4.

**A**

| Correlation Analysis c-KIT levels vs cell cycle status fractions of pre-HSC I population | | | | | |
|---|---|---|---|---|---|
| | **cKIT vs $G_0$-$G_1$** (red) | **cKIT vs early $G_1$** (grey) | **cKIT vs $G_1$/S** (yellow) | **cKIT vs S/$G_2$/M** (green) | **cKIT vs ratio red/green** |
| **r** | -0.8 | -0.5 | -0.4 | -0.1 | -0.7 |
| **R square** | 0.7 | 0.3 | 0.1 | 0.02 | 0.5 |
| **P value (two-tailed)** | 0.0004 | 0.04 | 0.2 | 0.7 | 0.004 |
| **P value summary** | *** | * | ns | ns | ** |

**B**

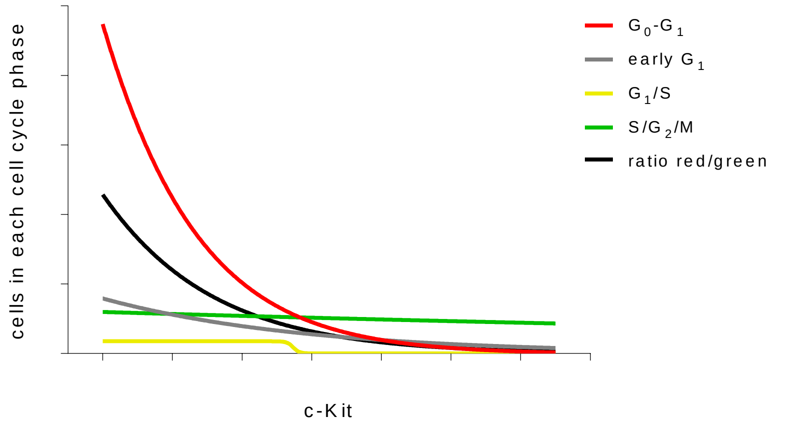


**Supplementary Table 2.** Related to Figure 4. **Correlation analysis of c-KIT levels with different cell cycle fractions within the pre-HSC Type I population.**

(A) E11.5 Pre-HSC Type I population from double transgenic Fucci embryos was analysed for cell cycle status and c-KIT levels by flow cytometry. Cell numbers extracted from the FlowJo analysis were used for the correlation analysis performed with GraphPad. The table shows the correlation coefficient (r), the r square and the p value (see Experimental Procedures). Pre-HSC Type I cells in $G_0$/$G_1$ inversely correlate with c-KIT levels similar to cells in early $G_1$ phase. Also the ratio of ($G_0$/$G_1$ cells) / (S/$G_2$/M cells) negatively correlate with c-Kit levels; therefore when c-KIT levels increase, the probability of $G_0$/$G_1$ cells within this fraction diminishes. On the contrary, there is no correlation between cells found in $G_1$/S and S/$G_2$/M phases and c-KIT levels and thus change in one variable (i.e. cell cycle phase) does not cause change in the other variable (i.e. c-Kit levels). (B) Nonlinear regression lines fit these data and depict the negative correlation between slowly cycling ($G_0$/$G_1$ and early $G_1$) cells and c-KIT levels.
